# On optimal temozolomide scheduling for slowly growing glioblastomas

**DOI:** 10.1093/noajnl/vdac155

**Published:** 2022-09-27

**Authors:** Berta Segura-Collar, Juan Jiménez-Sánchez, Ricardo Gargini, Miodrag Dragoj, Juan M Sepúlveda-Sánchez, Milica Pešić, María A Ramírez, Luis E Ayala-Hernández, Pilar Sánchez-Gómez, Víctor M Pérez-García

**Affiliations:** Neurooncology Unit, Unidad Funcional de Investigación de Enfermedades Crónicas (UFIEC), Instituto de Salud Carlos III (ISCIII), Madrid 28220, Spain; Instituto de Investigaciones Biomédicas I+12, Hosp. 12 de Octubre, Madrid 28041, Spain; Mathematical Oncology Laboratory (MOLAB), University of Castilla-La Mancha, Edificio Politécnico, Avda. Camilo José Cela 3. 13071 Ciudad Real, Spain; Institute of Applied Mathematics in Science and Engineering (IMACI), Castilla-La Mancha University, Spain; Neurooncology Unit, Unidad Funcional de Investigación de Enfermedades Crónicas (UFIEC), Instituto de Salud Carlos III (ISCIII), Madrid 28220, Spain; Instituto de Investigaciones Biomédicas I+12, Hosp. 12 de Octubre, Madrid 28041, Spain; Department of Neurobiology, Institute for Biological Research “Siniša Stanković”—National Institute of Republic of Serbia, University of Belgrade, Despota Stefana 142, 11060 Belgrade, Serbia; Instituto de Investigaciones Biomédicas I+12, Hosp. 12 de Octubre, Madrid 28041, Spain; Department of Neurobiology, Institute for Biological Research “Siniša Stanković”—National Institute of Republic of Serbia, University of Belgrade, Despota Stefana 142, 11060 Belgrade, Serbia; Neurooncology Unit, Unidad Funcional de Investigación de Enfermedades Crónicas (UFIEC), Instituto de Salud Carlos III (ISCIII), Madrid 28220, Spain; Mathematical Oncology Laboratory (MOLAB), University of Castilla-La Mancha, Edificio Politécnico, Avda. Camilo José Cela 3. 13071 Ciudad Real, Spain; Institute of Applied Mathematics in Science and Engineering (IMACI), Castilla-La Mancha University, Spain; Departamento de Ciencias Exactas y Tecnología Centro Universitario de los Lagos, Universidad de Guadalajara, Enrique Díaz de León 1144, Colonia Paseos de la Montaña, Lagos de Moreno 47460, Jalisco, Mexico; Neurooncology Unit, Unidad Funcional de Investigación de Enfermedades Crónicas (UFIEC), Instituto de Salud Carlos III (ISCIII), Madrid 28220, Spain; Mathematical Oncology Laboratory (MOLAB), University of Castilla-La Mancha, Edificio Politécnico, Avda. Camilo José Cela 3. 13071 Ciudad Real, Spain; Institute of Applied Mathematics in Science and Engineering (IMACI), Castilla-La Mancha University, Spain

**Keywords:** *in-silico* trials, mathematical oncology, optimal drug scheduling, temozolomide resistance, tumor phenotype

## Abstract

**Background:**

Temozolomide (TMZ) is an oral alkylating agent active against gliomas with a favorable toxicity profile. It is part of the standard of care in the management of glioblastoma (GBM), and is commonly used in low-grade gliomas (LGG). *In-silico* mathematical models can potentially be used to personalize treatments and to accelerate the discovery of optimal drug delivery schemes.

**Methods:**

Agent-based mathematical models fed with either mouse or patient data were developed for the *in-silico* studies. The experimental test beds used to confirm the results were: mouse glioma models obtained by retroviral expression of EGFR-wt/EGFR-vIII in primary progenitors from p16/p19 ko mice and grown *in-vitro* and *in-vivo* in orthotopic allografts, and human GBM U251 cells immobilized in alginate microfibers. The patient data used to parametrize the model were obtained from the TCGA/TCIA databases and the TOG clinical study.

**Results:**

Slow-growth “virtual” murine GBMs benefited from increasing TMZ dose separation *in-silico*. In line with the simulation results, improved survival, reduced toxicity, lower expression of resistance factors, and reduction of the tumor mesenchymal component were observed in experimental models subject to long-cycle treatment, particularly in slowly growing tumors. Tissue analysis after long-cycle TMZ treatments revealed epigenetically driven changes in tumor phenotype, which could explain the reduction in GBM growth speed. *In-silico* trials provided support for implementation methods in human patients.

**Conclusions:**

*In-silico* simulations, *in-vitro* and *in-vivo* studies show that TMZ administration schedules with increased time between doses may reduce toxicity, delay the appearance of resistances and lead to survival benefits mediated by changes in the tumor phenotype in slowly-growing GBMs.

**Key Points**

1. Larger dose spacings lead to better survival and toxicity in murine GBM models.
2. Virtual trial suggests improved survival in GBM patients by increasing dose spacing.
3. Long-cycle temozolomide treatments reveal epigenetically-driven changes in GBM phenotype.

**Importance of the Study**

*In-vivo* evidence is provided of improvements in survival, resistance, and toxicity from TMZ schemes with long rest periods between doses in slowly growing GBM mouse models. The results match hypotheses generated *in-silico* using a mathematical model incorporating the main biological features and fed with real patient data. An epigenetically driven change in tumor phenotype was also revealed experimentally, which could explain the reduction in GBM growth speed under the “long cycle” scheme. To determine the extent to which our results hold for human patients, large sets of simulations were performed on virtual patients. These *in-silico* trials suggest different ways to bring the benefits observed in experimental models into clinical practice.

Adult gliomas are the most common primary malignant tumors of the central nervous system. The new WHO (World Health Organization) classification distinguishes between IDH1/2 (isocytrate dehydrogenase 1/2) mutant gliomas, which include lower-grade gliomas (LGGs) (grade 2–3) and grade 4 IDH1/2 mut gliomas, and IDH1/2 wild-type glioblastomas (GBMs). GBMs are diagnosed at a later age (median 64) and have a dismal prognosis (15 months’ overall survival) despite the standard-of-care treatment, which consists of maximal surgical resection followed by radiotherapy (RT) plus concomitant and adjuvant chemotherapy (CT) with temozolomide (TMZ), an oral alkylating agent.^1,2^ TMZ is administered orally at a dose of 75 mg/m^2^ daily throughout RT, plus 6 cycles of maintenance TMZ 150–200 mg/m^2^ for 5 out of 28 days.^2^ The cytotoxicity of this drug is attributed to the addition of methyl temozolomide groups to DNA, and especially to the formation of O6-methylguanine (O6-meG) lesions and the subsequent formation of double-strand breaks during DNA replication, which requires cell division for the emergence of the cytotoxicity. As O6-meG can be removed by methylguanine methyltransferase (MGMT) in tumors expressing this protein, MGMT promoter methylation is considered a predictive biomarker of TMZ response in gliomas.^3^ Based on the short half-life of TMZ, it was suggested that high doses or repeated doses could improve its effect and reduce the capacity of cells to repair the DNA.^4^ However, dose-dense TMZ, with the administration of lower but continuous doses with the aim of depleting intracellular MGMT, did not show improved efficacy in newly diagnosed GBMs,^5^ and has shown only modest results in recurrent tumors^6–8^ at the cost of increased hematological toxicity.

Mathematical models describe real systems by abstraction and mathematical formalism. They may complement experimentation in providing a broader picture^9^ and suggest what the best RT, CT, or combination regimens might be, aiding the implementation of the treatment of cancers. Mathematical models of LGGs have been constructed in order to study the optimal delivery scheme of cytotoxic therapies.^10–12^ In slow-growth gliomas, such as LGGs, only a small percentage of cells is proliferating.^13^ It may be guessed that intensive therapies intended to deliver the maximum tolerated dose in the shortest possible time may be overkill for these tumors. Indeed, several authors have proposed that schemes with longer spacing between doses could produce better results than standard therapy in LGG patients.^10–12,14^ However, the simple mathematical models developed previously account only for a limited number of key biological processes. Notably, no detailed theoretical models, including realistic resistance mechanisms, have been considered previously. Also, it is still unknown whether the potential gain observed *in-silico* for LGGs would apply to GBMs as well. Although GBMs are considered as highly mitotic tumors (25% Ki67 labeling index, LI, on average), there is a broad range of Ki67% LI, with a not negligible percentage of GBMs (IDHwt) showing a low proliferative pro file.^15^ Independently of proliferation, the proneural (PN) to mesenchymal (MES) phenotypic transition observed in GBMs, either spontaneously or as a result of treatment (CT/RT), would limit the effect of TMZ as the tumor becomes more resistant.^16–18^ Also, there is a growing body of literature suggesting that the evolution of tumor cells to a fully drug-resistant state may often proceed through a reversible drug-tolerant phase,^19^ so-called persister cells.^20^ Rabé et al identified a population with persister characteristics under TMZ treatment of glioma cells.^21^ One may guess that longer spacing between cycles could delay the PN-MES transition, as well as allowing for persister cells to revert to their normal sensitive states.

The intention of this article was to set out a proof of concept supporting treatment regimes with longer times between doses of TMZ (hereafter referred to as protracted TMZ schedules, PTS) for IDH wild-type GBMs with different levels of proliferation. Our hypothesis was that PTS could lead to reduced appearance of persister cells. Moreover, reduced toxicity and increased tolerance were anticipated from increasing the rest period between cycles. To test these ideas, a stochastic mesoscopic discrete mathematical model (SMDMM) was first produced, including all relevant biological processes expected to play a role in the response of GBMs to TMZ. Next, PTS was studied *in-vitro* and in animal models and found to be in good agreement with the *in-silico* observations. The tissue of TMZ-responding allografts was then analyzed, and showed a striking change in tumor phenotype, with an increase in PN and a decrease in MES markers, driven by epigenetic changes, which could explain the reduction in tumor growth. Finally, exploratory virtual “clinical” trials were performed with *in-silico* tumors simulated with the SMDMM, to guide the implementation of the concept in clinical practice. The results indicated that survival is increased as spacing between doses becomes progressively larger, until a threshold is reached; spacings beyond this threshold would fail to improve survival.

## Methods

### Cell Lines and Cell Culture

The human GBM U251 cell line was purchased from American Type Culture Collection (ATCC, USA) and grown as recommended. Mouse SVZ cell lines were obtained by retroviral expression of EGFRwt or EGFRvIII and they were grown as previously described.^22,23^ Both models express GFP and luciferase as a reporter.^22,23^

### Production of Alginate Microfibers With U251 Immobilized Cells

Alginate microfibers with cells were produced by extrusion as described earlier.^24^ U251 cell lines were immobilized in alginate microfibers by the same procedure.

### Viability Study

The impact of 3 different TMZ (Sigma-Aldrich) (100 µM) treatment modalities (everyday treatments, X+1, and protracted (every 3 days, X+3; every 7 days, X+7)), starting from day 7, was determined by comparing the effects on cell viability, morphology, and aggregation using a CalceinAM (CAM)/propidium-iodide (PI) assay. Z-stack projections and quantitative estimation of the cell mass were analyzed using ImageJ software.

### *In-vitro* Treatments of Mouse Cells

SVZ EGFR wt/amp cells were incubated in the presence of TMZ (25 µM), which was supplemented 3 times: 1 day (X+1), 3 days (X+3), or 7 days (x+7) after the first dose. We have used this schedule based on the in vitro proliferation rate of these cells, which can be maintained for a maximum of 7 days, and we have used 3 days as intermediate data. In a different experiment, SVZ EGFR wt/amp cells were treated with TMZ (25 µM) and/or Azacytidine (AZA) (Sigma-Aldrich) (5 µm) for 7 days. For both experiments, cells were collected and lysed and subsequently analyzed by qRT-PCR, as described in Supplementary Methods.

### Intracranial Tumor Formation and Treatment *In-vivo*

Animal experiments were reviewed and approved by the Research Ethics and Animal Welfare Committee at “Instituto de Salud Carlos III” (PROEX 02/16), in agreement with the European Union and national directives. Intracraneal transplantation was done as previously described.^22,23^ Mice were treated with TMZ (10 or 50 mg/kg through intraperitoneal injection, i.p., with the schedules given in the various experiments). TMZ was dissolved in PBS+1% BSA, which was used to treat control animals. Animals were sacrificed when they showed symptoms of disease. At this final end-point, brains were dissected out and processed for cellular and molecular analysis.

### RNA Extraction and RT-PCR

RNA was extracted from human or mouse glioma cell pellets and tumor tissue. cDNA was synthesized and quantitative real-time PCR (qRT-PCR) was performed. The primers used for each reaction are indicated in Supplementary Table S1.

### *In-silico* Analysis

The Cancer Genome Atlas (TCGA) GBM dataset was accessed via UCSC xena-browser (https://xenabrowser.net) to extract proliferation gene expression levels. Classification into classical, mesenchymal, neural and proneural subtypes was retrieved from the TCGA GBM data set,^25^ together with gene expression values. Differences in gene expression between different groups were calculated using Student’s *t*-test.

### Statistical Analysis

The difference between experimental groups was assessed by paired *t-*test and one-way analysis of variance (ANOVA). For Kaplan-Meier survival curves, the significance was determined by the two-tailed log-rank test. All analyses were carried out with the GraphPad Prism 5 software. *P* values below .05 were considered significant (*P < .05; **P < .01; ***P < .001; ****P < 0.0001; n.s., not significant), both for mouse and simulated tumors. All experimental quantitative data presented are the means +/- SEM from at least 3 samples or experiments per data point.

### SMDM Model

An on-lattice SMDM model^26^ was adapted to simulate the longitudinal growth dynamics of GBM and its response to treatments *in-silico*. A comprehensive model description is provided in the Supplementary Information. This study included three basic cellular populations: proneural cells (either proliferative PNs or quiescent PNq), persister cells (P), and mesenchymal cells (either proliferative MESs or quiescent, MESq). The cell dynamics between different compartments is summarized in Supplementary Figure S4 (and in the Supplementary Information). It was assumed that PN cells may become MES cells, either directly, due to local vessel damage and hypoxia once the local cell density exceeds a critical threshold, or through a transient intermediate persister state induced by TMZ exposure. Both of these routes were associated with the emergence of TMZ resistance, as MES cells are assumed to be more resistant to TMZ than PN cells.

The methodology was first used to run simulations to explore the influence of the parameters on outcome, and to test the efficacy of the use of different dose spacings using murine parameters. Both fast and slow-growing virtual tumors were considered. Murine tumors were simulated without treatment (control), and treated with 3 TMZ doses, separated by 1, 4, 7, and 13 days. Human tumors were simulated without treatment, under standard TMZ therapy (6 cycles of TMZ given for 5 days, and resting periods of 3 weeks), and under 2 different courses of TMZ, increasing the spacing between doses. A first set of simulations was performed by increasing the rest periods between doses from 3 weeks to 9 weeks. Another set of computational studies was run by increasing the spacing between individual doses, while removing the rest periods. In particular, spacings of 8 and 12 days between doses were considered. Human and murine parameters were estimated from previous studies (see Supplementary Table S2) and our own datasets. Virtual human simulations were fed with real patient data to generate realistic tumors *in-silico*.

The simulator was implemented in Julia (version 1.1.1). Simulation file processing and graphics were undertaken in MATLAB (R2021a, MathWorks). Simulations were performed on two 2.4 GHz, 16-core, 192 GB memory Mac Pro machines. Computational cost per murine simulation ranged from 5 to 10 min, while for humans, the computational cost ranged from 20 to 50 min per simulation.

## Results

### Slow Growth Murine GBMs Benefitted From Increasing TMZ Dose Separation *In-silico*

**Figure 1.**
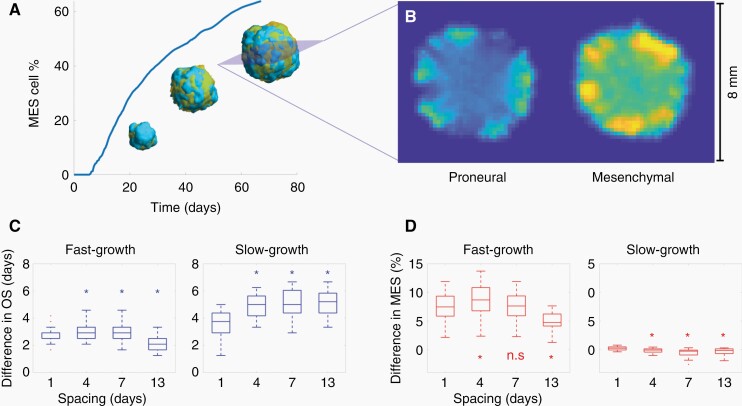
Mice tumor simulations predict an improvement in antitumor effect of TMZ in slow-growth GBMs. (A) Depiction of PN-MES transition in a single simulation, showing the evolution of MES cell abundance over time. 3D volumes are rendered at 50, 75, and 100% of simulation time (blue: PN cells; orange: MES cells). (B) Axial plane showing cell number per voxel in a simulated tumor at the end of simulation. Both PN and MES cell numbers belong to the same slice. (C) OS gain (in days) produced by TMZ dose spacing compared against control. 4-, 7-, and 13-day spacings were compared against 1 day spacing (Wilcoxon signed-rank test). (**D**) MES cell increase (percentage) compared against control. 4-, 7-, and 13-day spacings were compared against 1 day spacing (Wilcoxon signed-rank test, *P ≤ .05, n.s.=non-significant).

Virtual murine tumors were simulated as described in Methods, to explore the effect of different TMZ schemes in OS and MES content. A sustained increase in MES cell abundance was observed in untreated tumors (Figure 1A), reproducing the aforementioned PN-MES transition. These values are in agreement with real mouse data obtained in our *in-vivo* experiments. Notably, the tumor boundary was specially enriched in MES cells (Figure 1B).

Treatment with TMZ yielded an increase in OS for both fast- and slow-growth tumors (Figure 1C). This survival increase was higher for slow-growth tumors; moreover, enlarging the spacing between doses produced a better response in this kind of tumors. Regarding resistance, TMZ induced a significant increase in MES cell content in fast-growth tumors (Figure 1D). However, slow-growth tumors did not undergo such increase; most of them remained with the same amount of MES cells as control tumors, or even reduced their MES levels. This effect was more evident for long spacings.

A robust observation was that virtual mice with slow-growth murine tumors had the largest survival increase when increasing the spacing between doses *in-silico*. This benefit was preserved through the regions of the parameter space explored. Longer spacing between doses led to reduced mesenchymal component in the final tumors as compared to the 1-day spacing. Altogether, the simulations suggest that longer spacing between doses would be more effective against slow-growth gliomas, both in terms of OS and resistance development (Figure 1C and D).

### Effect of Protracted TMZ in Two Mouse Glioma Models With Different Proliferation Kinetics

To validate the hypothesis that protracted TMZ regimes could offer therapeutic advantages depending on the degree of proliferation of GBMs, mouse models were used, generated in our lab by overexpressing EGFRwt or EGFRvIII in p16/p19 ko subventricular zone (SVZ) progenitors. Both models generate gliomas in nude mice with a high penetrance and reproducibility. Notably, animals survive for two months after SVZ-EGFRwt cell intracranial injection, whereas SVZ-EGFRvIII tumors kill the animals much faster,^23^ in agreement with the higher aggressiveness attributed to the mutated isoforms of EGFR.^27,28^ Moreover, our previous analyses showed that tumors formed by SVZ-EGFRvIII cells were much more proliferative than those formed by SVZ-EGFRwt cells^23^ although both cell lines are equally sensitive to TMZ in vitro (Supplementary Figure 1A).

**Figure 2.**
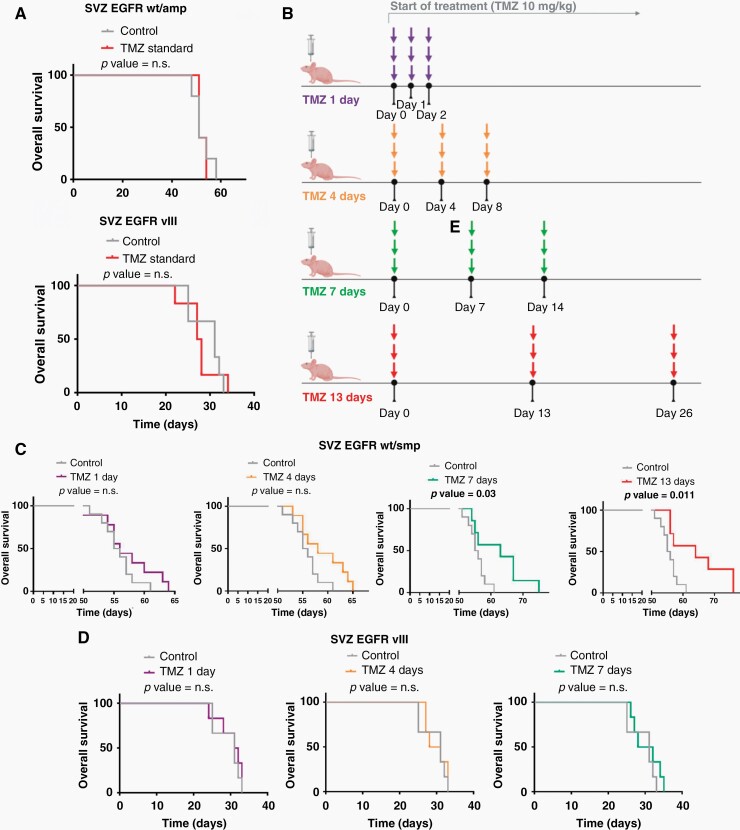
Increasing spacing between TMZ doses improves the anti-tumor effect of TMZ in the SVZ-EGFR wt/amp (lower growth rate) but not in the SVZ-EGFR vIII (faster growth speed) model. (A) Kaplan-Meier overall survival curves of mice that were orthotopically injected with SVZ EGFR wt/amp (top) or SVZ-EGFR vIII (bottom) cells and subsequently treated with intraperitoneal injections (5 days per week) of temozolomide (TMZ) (10 mg/kg per day) (*n* = 6). (B) Representative scheme of the different TMZ schedules studied. (C–D) Kaplan-Meier overall survival curves of mice that were orthotopically injected with SVZ-EGFR wt/amp (*n* = 9) (C) or SVZ-EGFR vIII (*n* = 6) (D) cells and subsequently treated with intraperitoneal injections of TMZ (10 mg/kg per day) following the different treatment protocols explained in (B), each of them represented by its assigned color.

Standard treatment of mouse glioma models with TMZ consisted of daily (5 days/week) i.p. injections of 10 mg/kg/day of the compound, which did not produce a survival benefit in animals bearing SVZ-EGFRwt or SVZ-EGFRvIII tumors (Figure 2A). Several TMZ regimes were then designed with three consecutive doses of three TMZ injections, separated by 1 day (X+1), 4 days (X+4), 7 days (X+7) or 13 days (X+13) (Figure 2B), although this last schedule could not be applied in the EGFRvIII model due to their faster growth. The graphs in Figure 2C show a clear reduction in SVZ-EGFRwt tumor growth in the X+7 and X+13 protracted schemes. Extending the interval between TMZ doses did not improve the response of SVZ-EGFRvIII bearing animals (Figure 2D). This result validates (at least in mice) the suggestion from the SMDMM that slowly-growing tumors are those that most benefit from protracted regimes.

### Increasing Spacing Between TMZ Doses Does not Increase Cell Death in the Tumors but Reduces the Expression of Persister-Related Genes

**Figure 3.**
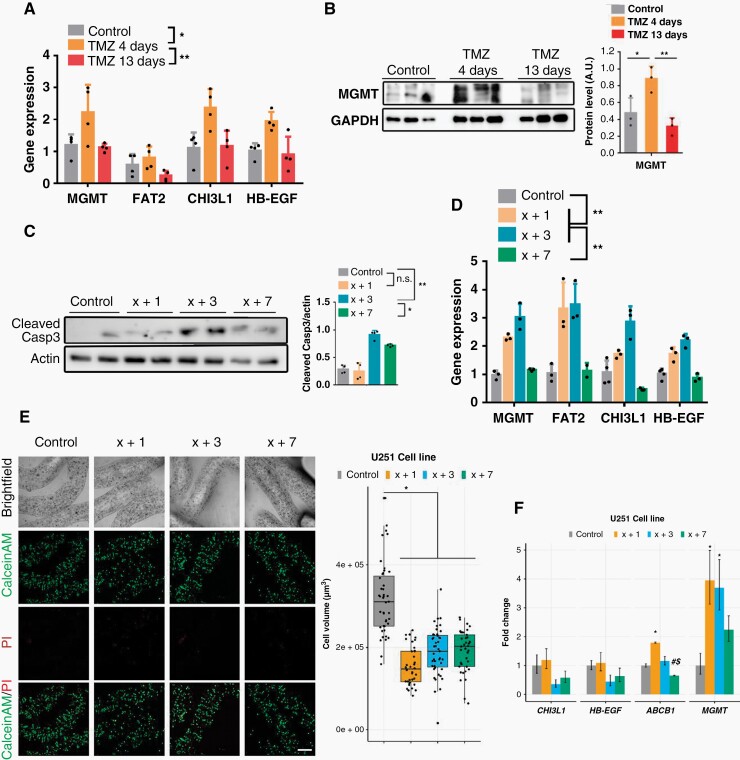
Increasing the spacing between TMZ treatments reduces the expression of persister-related genes *in-vivo* and *in-vitro*. (A) qRT-PCR analysis of persister-related genes in SVZ-EGFR wt/amp tumors from (Figure 2). *Actin* was used for normalization (*n* = 4). (B) Western blot (WB) analysis and quantification of the expression of MGMT in SVZ tumors from (A). GAPDH was used for normalization. (C) WB analysis and quantification of Active Capase3 in SVZ-EGFR wt/amp cells treated *in-vitro* with different TMZ schedules: control, TMZ 1 day, TMZ 3 days, and TMZ 7 days. *Actin* was used for normalization. (D) qRT-PCR analysis in SVZ-EGFR wt/amp cells with the different treatment protocols. *Actin* was used for normalization (*n* = 4). (E) Representative confocal microscopy images of U251 cells LIVE/DEAD labeled with CalceinAM/PI in alginate microfibers after 28 days when the treatments and cultivation of cells were completed. Quantification is shown as a box-plot on the right (*n* ≥ 3) Scale bar = 300 µm. (F) Relative gene expression levels of persister-related genes. *ACTB* was used for normalization (*n* = 3). *P ≤ .05, **P ≤ .01, n.s.=non significant. # indicates *P* < 0.05 statistical difference compared to corresponding X+1 treatments. $ indicates *P* < .05 statistical difference compared to corresponding X+3 treatments.

To understand the beneficial effect of PTS, tumor tissue in the SVZ-EGFRwt model was analyzed, comparing the control-treated with the X+4 (no response) and the X+13 (responsive) tumors. Notably, no changes were found in the tumor size in any of the schemes (Supplementary Figure 1B). In the X+13 scheme, a small decrease was observed in the number of proliferating cells (Supplementary Figure 1C), with no increase in the number of apoptotic cells (Figure 3A) compared to control tumors. However, in the X+4 scheme there was no change in proliferation, whereas an increase in the number of apoptotic cells was measured (Supplementary Figure 1D). These results suggest that the reduction in the tumor growth observed after X+13 protracted administration of TMZ is not mediated by an increase in the cell death response.

As previously mentioned, persister cells represent an intermediate phenotype arising before the development of TMZ resistance in GBMs.^21^ Notably, the expression of persister genes was induced in tumors that had been treated with the X+4, but not with the X+13, regime, as compared to untreated animals (Figure 3A). Interestingly, one of these genes is *MGMT*, whose expression is strongly associated with TMZ resistance.^29^ It was confirmed that MGMT protein was indeed being accumulated in the X+4 but not in the X+13 scheme (Figure 3B). These results suggest that extending the rest periods between TMZ treatments not only improved the anti-tumor effect of the drug, but also reduced the appearance of a persister state in the tumor cells.

In order to test whether the effect of protracted TMZ was cell-autonomous, SVZ-EGFRwt cells were treated with different schedules of TMZ *in-vitro.* An increase was observed in caspase 3 cleavage in the X+3 scheme (Figure 3C), accompanied by the accumulation of the expression of persister genes, including *MGMT* (Figure 3D). Notably, an extended period between TMZ doses (X+7 scheme) did not produce any of these effects (Figure 3C and D). These results suggest that the anti-tumor mechanism of protracted TMZ doses is the same *in-vivo* and *in-vitro* and does not depend on the tumor microenvironment. Moreover, we also treated SVZ-EGFRvIII cells with different schedules of TMZ *in-vitro*. As they did not respond to PTS, we expect to see no reduction of persister genes’ expression. Indeed, we found that expression of persister genes is increased in response to TMZ, but stays increased independently of the schedule used (compared to control; see Supplementary Figure S4).

To confirm these results in human cells, U251 cells, grown in conventional 2D conditions, were exposed to three doses of TMZ (100 µM), comparing daily (X+1) and 3-day (X+3) schedules (Supplementary Figure 6A). Both regimes were able to reduce the growth of the cells compared to the control, according to the change in doubling time (Supplementary Figure 6B). However, in both cases an increase in the expression of resistance-related markers in the TMZ treated cells was detected (Supplementary Figure 6C). In order to test the effect of PTS in conditions where the cells would have slower growth, U251 cells were immobilized in alginate microfibers and grown in 3D conditions. The cells were then exposed to three doses of TMZ (100 µM), comparing daily (X+1), 3-day (X+3), and 7-day (X+7) schedules (Figure 3E). In all cases, TMZ reduced the growth of the cells (Figure 3F). Notably, X+7 decreased the expression of persisters-related genes in TMZ treated cells (Figure 3F). These results suggest that enlarging the intervals between doses could reduce the appearance of a persister phenotype *in-vitro* in human glioma cells, at least in conditions of reduced proliferation.

### Protracted TMZ Induced a Change in the Phenotype of Slowly Proliferating GBMs, Mediated by Epigenetic Changes

**Figure 4.**
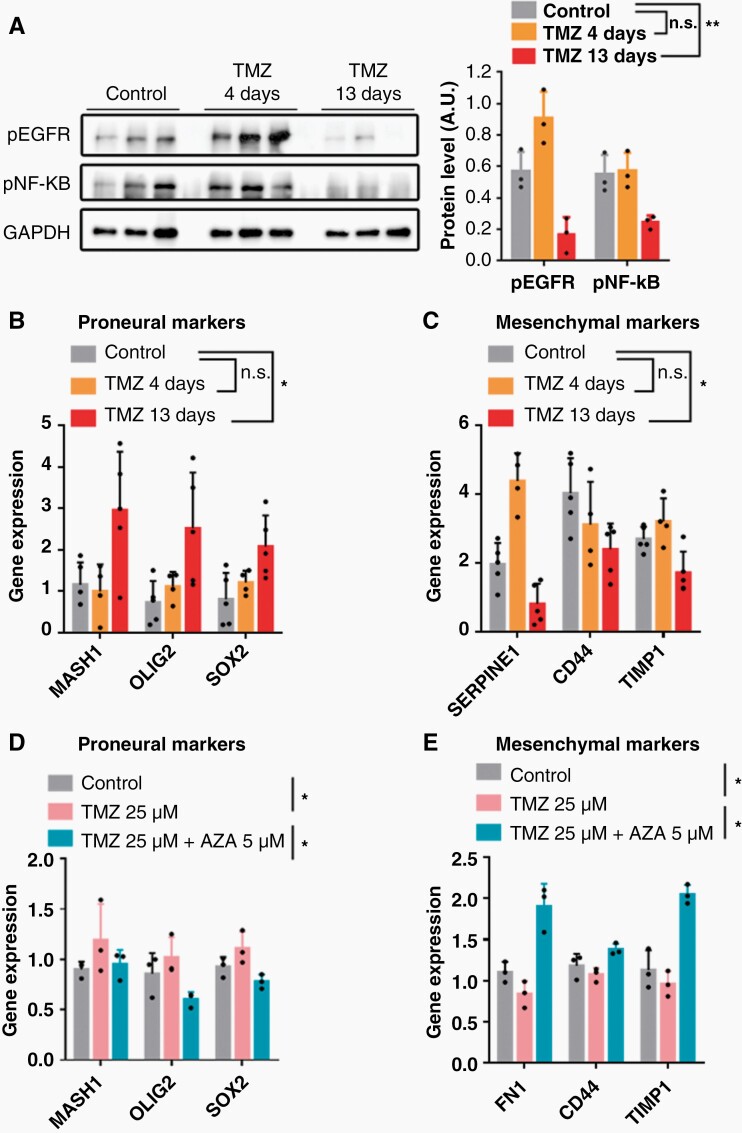
Change of glioma phenotype after different TMZ treatment schedules. (A) Western blot analysis and quantification of phosphorylated EGFR (pEGFR) and NF-kB (p65) (pNF-kB) in SVZ EGFR wt/amp tumors from (Figure 2). GADPH was used as a loading control (*n* = 3). (B–C) qRT-PCR analysis of Proneural (B) and Mesenchymal subtype (C) related genes in SVZ EGFR wt/amp tumors from (A). *Actin* was used for normalization. A paired *t*-test to evaluate the 3 markers of each genetic signature together was performed (*n* = 4). (D-E) Analysis of the expression of Proneural (D) and Mesenchymal markers (E) transcription by qRT-PCR in SVZ EGFR wt/amp cells cultured in the presence of 25 µM TMZ, with or without azacytidine (AZA) (5 µM). *Actin* was used for normalization. A paired *t*-test to evaluate the 3 markers of each genetic signature together was performed (*n* = 3). *P ≤ .05, **P ≤ .01, n.s. = non significant.

Our previous characterization of the SVZ-EGFRwt model shows that these cells express MES features.^22,23^ Interestingly, an *in-silico* analysis of two proliferation markers, PCNA and MKI67, was performed, and it was found that MES gliomas expressed the lowest levels of these genes (Supplementary Figure 7A), suggesting that tumors with this phenotype are less proliferative than the other 2 subtypes (classical (CL) and PN). Our previous data have shown that the MES profile of SVZ-EGFRwt tumors depends on the activation of the EGFR/NFkB signaling pathway. Notably, EGFR has been associated with TMZ resistance in gliomas.^30^ The activation status of this receptor in SVZ-EGFRwt tumors treated with TMZ was therefore explored, both in responsive and non-responsive schedules. A clear downregulation of the levels of phosphorylation of EGFR and NF-kB was observed in the X+13 tumors (Figure 4A). In tumors from the X+4 regime there was an increase in phospho-EGFR, although no significant changes were observed in the amount of phospho-NFkB (Figure 4A), a MES driver in gliomas.^31^ The changes observed in the X+13 responsive tumors were paralleled by an increase in the expression of several PN markers (Figure 4B) and the downregulation of the transcription of MES genes (Figure 4C), compared to control tumors. These results suggest that less intensive TMZ schedules might be reducing the aggressiveness of GBMs by inducing a MES to PN phenotypic change. Notably, no changes in the amount of phosphorylation of EGFR or NF-kB were observed in TMZ-treated SVZ-EGFRvIII tumors compared to controls (Supplementary Figure 7B), reinforcing the lack of response of these gliomas to the drug.

To study the anti-tumor mechanisms of TMZ more deeply, SVZ-EGFRwt cells were incubated *in-vitro* in the presence of TMZ (25 μM) for 8 days. As noticed in tumors treated with TMZ, an increase in the expression of PN markers was observed (Figure 4D). Notably, this effect was reverted in the presence of the DNA-methyltransferase inhibitor 5-aza-2’deoxycytidine (AZA) (Figure 4D), suggesting that TMZ might be inducing the expression of these genes by a shift in the DNA methylation pattern, previously proposed as a mechanism of action of this drug in glioma cells.^32^ A decrease in the expression of MES genes was also noticed, which did not occur in the presence of AZA (Figure 4E). Therefore, it was hypothesized that the anti-tumor effect of protracted schemes of TMZ might be mediated, at least in part, by a MES-to-PN transition of the tumor cells induced by epigenetic changes, the opposite of what would normally happen during tumor progression.^33^ This would increase the effect of giving more time to persister cells to revert their phenotypes to the PN phenotype.

### TMZ Dose can be Increased in a Protracted Scheme to Enhance the Anti-Tumor Effect and Reduce Toxicity

**Figure 5.**
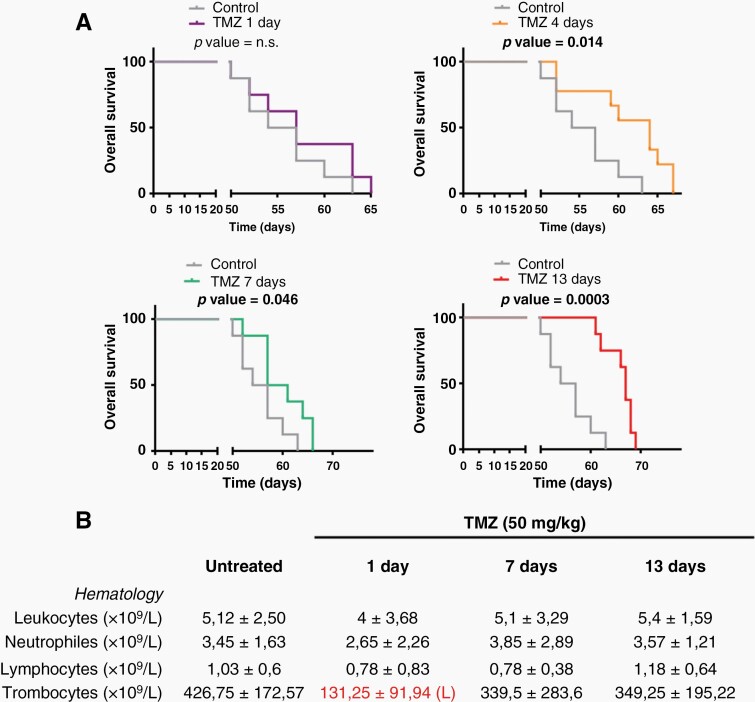
Reduced toxicity of protracted TMZ schemes. (A) Kaplan-Meier overall survival curves of mice that were orthotopically injected with SVZ EGFR wt/amp cells and subsequently treated with intraperitoneal injections of a higher dose of TMZ (50 mg/kg per day) following the different TMZ protracted schemes (*n* = 9). (B) Hematology results in mouse blood samples from (A).

One of the potential benefits of long spacing between TMZ cycles could be a reduction in the toxicity of the drug, which may perhaps allow the CT dose to be increased. To test this hypothesis, the amount of TMZ administered to the animals after the intracranial injection of SVZ-EGFRwt cells was increased to 50 mg/Kg/day. The same schemes for TMZ treatment were used as in Figure 2, and confirmed that the longest period between cycles was the most effective in reducing tumor growth (Figure 5A). The day after the last TMZ cycle, blood was collected from the animals to perform a white-cell count. One of the most common adverse effects of chemotherapy with TMZ is myelosuppression, including thrombocytopenia and leukopenia.^34^ Indeed, a reduction was observed in all the numbers in the X+1 regime that reached a statistically significant value for the decrease in thrombocytes (Figure 5B). Notably, extending the period between doses was able to revert to normal the leukocyte and thrombocyte counts (Figure 5B), indicating that the toxicity of TMZ was reduced, even though the anti-tumor effect was increased in comparison to the lower dose regime.

### Virtual Clinical Trials Suggest How to Translate the Results in Experimental Models to Human Patients

**Figure 6.**
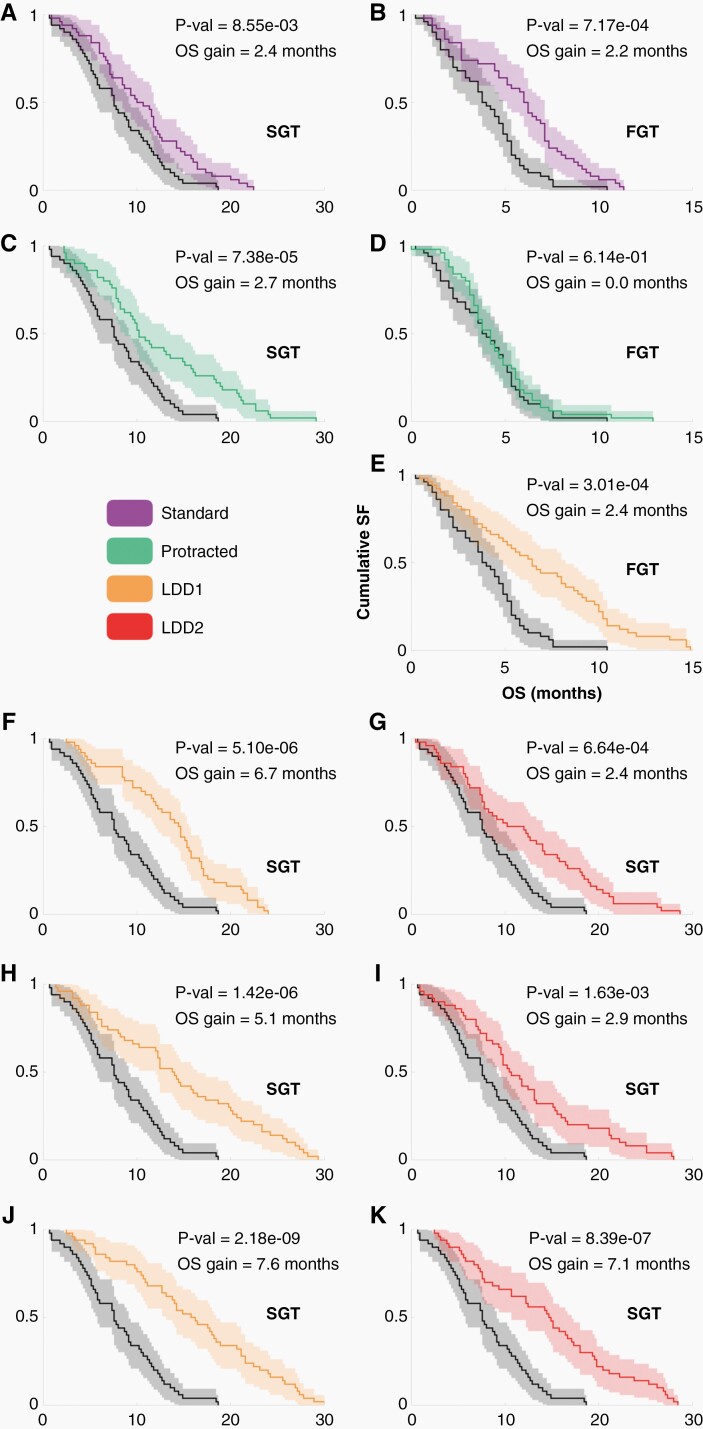
Kaplan-Meier survival curves for PTS *in-silico*. Standard therapy consists of 6 cycles of TMZ, with 5 doses per cycle, and 23 days of resting period. Protracted therapy consisted of standard therapy, with a resting period of 9 weeks. Low-dose-density therapy 1 (LDD1) correspond to doses separated by 8 days without any rest periods. Low-dose-density therapy 2 (LDD2) consisted of individual doses spaced by 12 days without rest periods. B, D, and E depict fast-growing tumors (FGT), while the other panels depict slow-growing tumors (SGT). Tumors in panels from A to G received 6 cycles of their corresponding therapy. Tumors in H and I received 12 cycles, while tumors in J and K received 18 cycles.

To extend previous *in-vivo* findings, many sets of virtual clinical trials were performed, based on the SMDMM with human parameters. The purpose is to observe the effect of increasing dose spacing on the response of the simulated tumors. Therefore, neither RT nor surgery have been implemented, so as to be able to compare the isolated effect of TMZ between the different spacings. The potential effect of PTS on tumor growth dynamics was assessed by (i) enlarging the rest periods between cycles, and (ii) testing long separation times between individual doses without rest periods (Figure 6). Simulated tumors were separated in slow-growing and fast-growing GBMs. All *in-silico* patients were given 30, 60, or 90 doses of TMZ 7 weeks after diagnosis (depending on therapy regime).

Standard TMZ schemes (5 consecutive doses, rest period of 3 weeks) showed a beneficial effect in terms of survival for both tumor types (Figure 6A and B), with a median survival difference of nearly 2 months, in line with clinical experience.^5^ Increasing the rest period to 9 weeks improved survival for slow-growth tumors, with an increased number of longer survivors (Figure 6C). Fast-growing tumors did not benefit from the increase in spacing between doses (Figure 6D), in line with our observations in animal models.

On the other hand, all trial branches benefited from increasing time intervals between doses (without resting periods), both for slow and fast-growing tumors. As the number of cycles given was increased, differences in median survival increased remarkably.

## Discussion

TMZ is the standard of care for newly diagnosed GBM, but the effect of this alkylating agent is schedule-dependent.^35^ Genetic or acquired resistances to TMZ can easily develop, and a strict regimen must be followed for a favorable result to be obtained.^36,37^ The design of cytotoxic CT and RT schedules is typically based on the basic principle of delivering the maximum tolerated dose in the minimum time possible (MTDMT) to avoid potential tumor repopulation in the periods without treatment. Although this is certainly the way to go when CT is intended as a curative treatment, it is not obvious that it would be the best strategy when it is known that treatment can only control disease for a limited time.

Intensification of TMZ delivery schemes has been studied for either newly diagnosed^5^ or recurrent^7,8^ high-grade gliomas without positive results on OS and with increased toxicity.^38^ However, no previous studies have considered effective dose-reduction schemes with longer time spacing between treatments.

This work studies, *in-vitro*, *in-vivo*, and *in-silico* using mathematical models, the main factor limiting the effectiveness of treatment in GBMs: the development of resistance. Resistance acquisition in the SMDMM was assumed to be due to the PN-MES transition, and persister induction by TMZ. Accounting for resistances led to improved survival and reduced resistance when increasing the interval between doses in virtual mice. The reduction of resistant cells stemmed from the fact that longer spacing between doses allowed persister cells to revert their phenotypes to PN. Clearly, when persistence time is longer than the spacing between doses, persister cells receive additional TMZ doses and the emergence of resistance is triggered. Very interestingly, for slow-growing tumors, as TMZ kills a fraction of both PN and MES cells, the resistance level at the end of the simulation was observed to be smaller than its control counterpart, as the spontaneous PN-MES transition is being reduced due to TMZ killing PN cells. Thus, increasing the spacing between doses provides the same effect as a reverse MES-PN transition. This setup was translated to the real world, both in *in-vitro* and *in-vivo*. Results in mice were in accordance with model suggestions: slow-growing tumors benefited most from PTS. In fact, fast-growing tumors did not show an improvement in OS, as expected.

A phenotypic change in slowly growing tumors subject to increased spacing between doses was observed *in-vivo*, with a reduction in the levels of phosphorylated EGFR and NF-κB, is associated with a MES-to-PN switch, as recently shown.^22^ This transition could explain the reduced aggressiveness of the tumors after long-cycle TMZ treatment, which does not seem to depend on changes in proliferation and/or survival of tumor cells. Notably, our data suggest that this MES to PN switch does not depend on the tumor microenvironment and can be reverted in the presence of AZA, the known epigenetic regulator. Changes in DNA methylation have already been associated with the response to TMZ in a time and dose-dependent manner.^32^ Moreover, it has been previously shown that the persister state is also linked with alterations in the levels of histone acetylation and with chromatin remodeling processes.^21^ Therefore, epigenetic changes might be responsible for the appearance of resistances, but also for some of the anti-tumor effects of TMZ, all linked to alterations in the transcriptomic profiles of GBMs. Our results might explain why extensive TMZ treatment did not alter the survival of PN gliomas, but was beneficial for the more aggressive MES subtype.^39^ Anyhow, these results not only emphasize the potential clinical relevance of PTS for slowly growing GBMs, but also indicate that the biological assumptions taken in the model of action of this compound are not far-fetched, and should be explored in deeper detail to keep improving our knowledge about GBMs and the best way to treat them.

The experimental evidence shown here supports the fact that PTS could be beneficial for GBM patients in terms of survival, resistance, and toxicity so far. The output yielded by the virtual clinical trial agreed with the experimental results obtained in this work. Standard TMZ therapy showed a moderate improvement in terms of survival, in line with clinical experience. Fast-growing tumors did not benefit from increasing the rest periods between cycles, but they did benefit from enlarging the spacing between doses. Slow-growing tumors benefitted not only from every alternative therapy scheme, but also from an increase in the number of cycles given due to the reduction in the MES component, and thus in tumor resistance. This points to alternative schemes that would allow for more TMZ doses to be given, while keeping resistances stable and with lower toxicity. There were several interesting implications from these virtual trial studies. The first was that extending the rest period between 5-dose cycles from 3 to 9 weeks showed a significant improvement in survival for patients with slow-growing GBMs, suggesting an easy-to-apply upgrade in the standard of care. The second was that there may be room for optimizing TMZ schedules in GBMs, as the best improvements in survival came from schemes without rest periods, and 8–12 days between doses. Due to their lower dose density, these schemes could be less harmful in terms of toxicity and, following both virtual and experimental results, a reduced resistance should also be expected, making them an alternative option for clinical implementation.

It is reasonable to expect that the results obtained in this work are generalizable to IDH-mut gliomas, and LGGs in general. Their slow growth and low proliferation rate would give much more scope for finding optimal spacings when treating with TMZ. In fact, there are theoretical studies that support this conclusion. Different studies based on mathematical models have argued that cytotoxic therapies with larger time intervals between doses could provide survival benefits in LGGs.^10–12^ However, these mathematical models are based on saturable growth models, where tumors proliferate less on average as they grow larger, and the phenomenon is lost when exponential growth models are considered.^12^ Moreover, the biological assumptions on which they are based are simple, and do not take into account phenomena such as persister-mediated resistance, or the phenotypic PN-MES transition. Studies integrating more complex theoretical models, as well as appropriate experimental models, are needed. The absence of the latter is another constraint, which is why the case of LGGs has not been explicitly considered in this work. We hope to develop suitable mathematical and experimental models in the future that will enable us to replicate this work, but for IDH-mut gliomas, to assess whether they also benefit from increased spacing between TMZ doses, and to which extent.

In conclusion, our combination of *in-silico* simulations, *in-vitro* and *in-vivo* studies showed that TMZ administration schedules with increased time spacing between doses may reduce toxicity, delay the appearance of resistances and lead to survival benefits mediated by changes in the tumor phenotype, which was especially important for slowly growing gliomas. The experimental results were extended to human patients showing different ways to improve survival based on the same concept.

## Supplementary Material

vdac155_suppl_Supplementary_Material

vdac155_suppl_Supplementary_Table_S1

vdac155_suppl_Supplementary_Table_S2
