# Supplementary material for: On optimal temozolomide scheduling for slowly growing glioblastomas
Source: Neurooncol Adv. 2022 Sep 27;4(1):vdac155. doi: 10.1093/noajnl/vdac155 (PMC9616068; doi:10.1093/noajnl/vdac155)
Supplement: vdac155_suppl_Supplementary_Material [file vdac155_suppl_supplementary_material.docx]

**On optimal temozolomide scheduling for slowly growing glioblastomas**

Berta Segura-Collar^1,2#^, Juan Jiménez-Sánchez^3,4#^, Ricardo Gargini^1,2#^, Miodrag Dragoj^5^, Juan M. Sepúlveda^2^, Milica Pešić^5^, María A. Ramírez^1^, Luis E. Ayala-Hernández^3,4,6^, Pilar Sánchez-Gómez^1*^, Víctor M. Pérez-García^3,4*^.

1. Neurooncology Unit, Unidad Funcional de Investigación de Enfermedades Crónicas (UFIEC), Instituto de Salud Carlos III (ISCIII), Madrid, Spain.

2. Instituto de Investigaciones Biomédicas I+12, Hosp. 12 de Octubre, Madrid 28041, Spain.

3. Mathematical Oncology Laboratory (MOLAB), University of Castilla-La Mancha, Edificio Politécnico, Avda. Camilo José Cela 3. 13071 Ciudad Real, Spain.

4. Institute of Applied Mathematics in Science and Engineering (IMACI), Castilla-La Mancha University, Spain.

5. Department of Neurobiology, Institute for Biological Research "Siniša Stanković" - National Institute of Republic of Serbia, University of Belgrade, Despota Stefana 142, 11060 Belgrade, Serbia*.*

6. Departamento de Ciencias Exactas y Tecnología Centro Universitario de los Lagos, Universidad de Guadalajara, Enrique Díaz de León 1144, Colonia Paseos de la Montaña, Lagos de Moreno 47460, Jalisco, Mexico.

^#^ Co-first authors

* Co-senior authors

**Running title:** Optimal temozolomide scheduling for slowly growing glioblastomas

**Corresponding authors:**

Víctor M. Pérez-García. Mailing address: Edificio Politécnico. Universidad de Castilla-La Mancha. Avenida de Camilo José Cela 3. Phone: +34-926295435. E-Mail: [victor.perezgarcia@uclm.es](mailto:victor.perezgarcia@uclm.es)

Pilar Sánchez-Gómez. Mailing address: Instituto de Salud Carlos III. Crtra. Majadahonda-Pozuelo Km2. Phone: +34-918223265. E-Mail: [psanchezg@isciii.es](mailto:psanchezg@isciii.es)

**Funding:** This research was funded by the James S. Mc. Donnell Foundation (USA) 21st Century Science Initiative in Mathematical and Complex Systems Approaches for Brain Cancer (Collaborative award 220020560, doi:10.37717/220020560); Ministry of Education, Science and Technological Development, Republic of Serbia (ref. number 451-03-9/2021-14/200007); Ministerio de Ciencia e Innovación and FEDER funds, Spain (grant number PID2019-110895RB-I00, doi: 10.13039/501100011033 to VMP-G, and RTI2018-093596 to PS-G); and Universidad de Castilla-La Mancha (grant number 2020-PREDUCLM-15634 to JJ-S).

**Conflict of interests:** The authors declare no competing interests.

**Authorship:** Study design and analysis: PS-G, JJ-S, JS, MP, VMP-G. Writing and review of manuscript: MP, JJ-S, BS-C, RG, PS-G, JS, VMP-G, MAR, LEAH. Research: BS-C, MD, RG, JJ-S. Mathematical modeling: JJ-S, VMP-G, LEAH. Software: JJ-S. Murine models: BS-C, RG, PS-G, MAR. Cell cultures: MD, MP. Project supervision: PS-G, VMP-G. Funding: PS-G, VMP-G. All authors revised and approved the manuscript.

**Word count:** 4527**SUPPLEMENTARY INFORMATION**

**Cell lines and cell culture**

Human GBM U251 cell line was cultured in Dulbecco’s Modified Eagle Medium (Biological Industries, USA) supplemented with 10% fetal bovine serum (Sigma-Aldrich, Germany), 2 mM glutamine (Sigma-Aldrich, Germany), 5,000 U/ml penicillin, and 5 mg/ml streptomycin (GibcoTM, Thermo Fisher Scientific, United States). Cells were cultivated at 37°C in a humidified 5% CO_2_ atmosphere and passaged twice a week after reaching 80-90% confluence using 0.25% trypsin/EDTA.

Mouse SVZ cell lines were obtained by retroviral expression of EGFRwt or EGFRvIII (retroviral vectors pBabe-EGFR wt (#11011) and MSCV-XZ066-GFP-EGFR vIII (#20737), respectively), and grown in primary neural stem cell cultures obtained from the subventricular zone (SVZ) of p16/p19 ko mice^1^. After infection, the cells were injected into nude mice, and the tumors that grew were dissociated and the lines SVZ-EGFRwt/amp and SVZ-EGFRvIII were established^2^.

Briefly, both models were maintained in stem cell medium; Neurobasal (Invitrogen) supplemented with B27 (1:50) (Invitrogen); GlutaMAX (1:100) (Invitrogen); penicillin-streptomycin (1:100) (Lonza); 0.4% heparin (Sigma-Aldrich); and 40 ng/ml EGF and 20 ng/ml bFGF2 (Peprotech). For dissociation and passage Accumax (ThermoFisher) was used.

**Viability study**

SVZ cells (EGFRwt or EGFRvIII) were incubated in the presence of increasing amounts of TMZ for 3 days and viability was measured with Alamar Blue. There were no significant differences in cell viability in response to TMZ between both cell lines (Supplementary Figure S1A). From this experiment we chose a dose of 25 μM of TMZ for the in-vitro experiments performed later, due to its small effect on cell viability. This sensitivity analysis was also performed over U251 cells (data is shown in Supplementary Figure S2).

To test whether different TMZ schedules could affect cell viability, we quantified the number of cells after TMZ treatment (under control, 1-day, 3-days and 7-days spacing schedules), and we observed that cell number did not significantly change between different schedules (Supplementary Figure S3).

**Production of alginate microfibers with U251 immobilized cells**

Briefly, 4 × 10^6^ cells/ml were mixed with a 2 % w/v Na-alginate solution to obtain final concentrations of 1.5% w/v Na-alginate. The Na-alginate solution with cells was manually extruded through a blunt edge stainless steel 25G needle immersed in the gelling bath (3 % w/v Ca(NO_3_)_2_ x 4H_2_O). Due to the exchange of Na^+^ with Ca^2+^, the liquid stream solidified in the gelling bath, thus forming insoluble microfibers. The microfibers were left in the bath for 15 min in order to complete gelling and were then washed with medium. After cell immobilization, 0.5 g of alginate fibers were distributed into a T25 flask and cultured for 28 days without passage in 13 ml of MEM medium. 50% of the medium was changed twice a week.

Regarding viability, U251 cells immobilized in alginate microfibers were cultured for 28 days and stained using CAM/PI as a LIVE/DEAD staining. Alginate microfibers containing cells were incubated for 45 min at 37 °C in medium with CAM in a final concentration of 4 μM while PI was added to a final concentration of 5 μM. Fluorescence microscopy images were taken using a Leica TCS SP5 II Basic confocal laser-scanning microscope (Leica Microsystems CMS GmbH; Germany), visualizing live (green) and dead (red) cells at every z-axis encompassing the alginate microfiber.

**Intracranial tumor formation and treatment *in-vivo***

Intracranial transplantation to establish orthotopic allografts was performed injecting 300,000 cells (resuspended in 2 μl of culture cell medium) with a Hamilton syringe into athymic Nude-Foxn1nu brains (Harlan Iberica). Female mice (2-3 months of age) were used, 7 to 10 animals per group. The injections were made into the striatum (coordinates: A–P, −0.5 mm; M–L, +2 mm, D–V, −3 mm; related to Bregma) using a Stoelting Stereotaxic device.

**Immunohistochemical (IHC) staining**

Tumor samples were fixed in 10% formalin overnight, dehydrated through a series of graded ethanol baths and then infiltrated with paraffin. Then, 2.5 µm-thick sections were obtained in a microtome and then sections were rehydrated and permeabilized (1% triton X-100). Antigen retrieval was performed with Citrate Buffer (10 mM, pH 6) in a pressure cooker (2 min). Endogenous peroxidase inhibition and blocking with normal horse serum was also carried out before the incubation with primary antibodies (anti-rabbit caspase3, 1:100, Cell signaling #9662), anti-mouse ki67, 1:100 Dako #M7248) (overnight, 4 ºC) and biotinylated secondary antibodies (HRP anti-mouse and HRP anti-rabbit, 1:200, GE Healthcare (2h at room temperature). Target proteins were detected with the ABC Kit and the DAB kit (Vector Laboratories).

**Western Blot analysis**

For protein expression analysis, mouse tumor tissue was processed by mechanical disruption in a lysis buffer (Tris–HCl pH 7.6, 1mMEDTA, 1mMEGTA, 1% SDS, and 1% Triton X-100) followed by heating for 15 min at 100ºC. Protein content was quantified by using a BCA Protein Assay Kit (Thermo Fisher Scientific). Approximately 30 µg of proteins were resolved by 10% or 12% SDS-PAGE, and these were then transferred to a nitrocellulose membrane (Hybond-ECL, Amersham Biosciences, Little Chalfont, UK). The membranes were blocked for 1 h at room temperature in TBS-T (10 mM Tris–HCl (pH 7.5), 100 mM NaCl, and 0.1% Tween-20) with 5% skimmed milk, and then incubated overnight at 4 ºC, with the corresponding primary antibody (mouse anti-MGMT 1:1000, BD Biosciences, #557045), mouse anti-GAPDH (1:1.500, Santa Cruz Biotechnology #sc-47724), rabbit anti-pTyr1068-EGFR (1:1.000, Cell Signaling #3777) and rabbit anti-phospho-NF-kB p65 (Ser536) (1:1000, Cell Signaling #3033) diluted in TBS-T. After being washed 3 times with TBS-T, the membranes were incubated for 2 h at room temperature with their corresponding secondary antibody (HRP-conjugated anti-mouse (#NA931) or anti-rabbit (#NA934), Amersham Biosciences) diluted in TBS-T.

**RNA extraction and RT-PCR**

Regarding human glioma cells, alginate microfibers with U251 cells were dissolved and cells were released after 28 days of incubation. To release cells, alginate microfibers were dissolved in 0.5 mM EDTA for 10 min at 37°C. Total RNA was extracted from control and treated group of cells. The extractions were carried out using Tri Reagent Solution (Invitrogen LifeTechnologies, USA) according to the manufacturer’s instructions. cDNA was synthesized using 2 μg total RNA and High-capacity cDNA reverse transcription kit (Applied Biosystems, USA) according to the manufacturer’s instructions.

For mouse samples, brain tumors were dissected out after the mouse sacrifice and fresh frozen. Alternatively, mouse cells grown *in-vitro* were collected and fresh frozen. RNA was extracted from the tissue or the cells using the RNA isolation Kit (Roche). Total RNA (1μg) was reverse transcribed with PrimeScript RT Reagent Kit (Takara).

Quantitative real time PCR was performed using the Light Cycler 1.5 (Roche) with the SYBR Premix Ex Taq (Takara). All experiments were performed in triplicate and relative gene expression levels were analyzed by the 2−ddCt method^3^.

**MGMT promoter methylation status**

The amount of MGMT protein was measured in SVZ-EGFRwt and SVZ-EGFRvIII tumors as a final readout of the methylation status of MGMT promoter, to assess whether its expression is uniform across mouse brains coming from different animal models. Protein was extracted from nude mouse brains and from both SVZ-EGFRwt and SVZ-EGFRvIII mouse brains, and a Western Blot analysis was performed. Quantification shows that MGMT is present in both types of tumors to a similar level compared with normal brain tissue (Supplementary Figure S5).

**Discrete mathematical model**

An adapted version of the on-lattice agent-based mesoscopic model^4^ was used to simulate GBM longitudinal growth dynamics and its response to the treatment in-silico. The basic cellular agents were clonal populations that could gain or lose cells through the different biological processes incorporated into the mathematical model: mitosis, migration, cell death or trait variations due to phenotypic changes. Cells in the same population behaved in the same way, except for intrinsic noise resulting in stochastic transitions. At each time step, cells could proliferate, die, migrate and/or change their phenotype mimicking GBMs’ behaviour. Proliferation, death and migration within each population were implemented as in previous works^4^. Tumor dynamics were simulated in-silico on a spatial domain discretized on a rectangular grid of voxels (volume units). Dynamics were voxel-specific depending on each voxel and neighbouring voxel occupation. In this work we incorporated three basic cellular populations: proneural cells (either proliferative PNs or quiescent PNq), persister cells (P), and mesenchymal cells (either proliferative MESs or quiescent, MESq). Aiming to reproduce the observed characteristics of both PN and MES phenotypes, we searched for the ‘advantages’ parameters providing realistic tumors in terms of PN-MES transition. After this analysis, proneural cells were assumed to grow faster than mesenchymal cells (25% faster), while mesenchymal cells migrated faster (twice as fast) and were assumed to be less affected by TMZ, to capture their increased resistance.

Since nor in mice models neither in in-vitro experiments there is time for mutational events to play a substantial role in the cellular kinetics, we assumed cells in each compartment to be clonal. PNs and MESs cells were assumed to proliferate, migrate, die or become quiescent with a probability μ_sq_. PNq and MESq cells did neither proliferate nor die, but they were allowed to migrate and revert their phenotypes to the proliferative state with probability μ_qs_. Tumor growth rates and proliferation levels (Ki67) are implicitly determined by the interplay between these two rates.

Once a voxel reached a cell number above 70% of its carrying capacity, PNs cells could change their phenotypes to MESs with a rate μ_PT_, due to the effect of local vessel damage and hypoxia. Under exposure to TMZ, PNs cells could either die or enter a persister (P) state with a rate μ_sp_. Cells in P state were allowed to migrate, but neither proliferate nor die. P was assumed to be a reversible intermediate transient state prior to gaining resistance to therapy. If exposure is prolonged, P cells switch their phenotypes to a MESs state with a rate μ_pr_, thus becoming resistant. However, halting exposure to therapy was assumed to allow P cells, but not MES cells, to go back to PNs state at a rate μ_ps_. MESs cells were assumed to be less sensitive to TMZ and could proliferate, migrate, die, or switch to a quiescent state (MESq). MES cells’ transition rates between proliferative and quiescent states are the same as those of PNq cells. Those transitions between the different compartments are summarized in Figure S4.

Each spatial voxel may contain several cells belonging to the compartments described before with an upper limit denoted to as local carrying capacity *K*. At a given time step, each cell attempts to perform the different basic processes. These processes can be regarded as two-outcome events, being the possible outcomes success or failure. Therefore, a single cell attempting a given process can be considered a Bernoulli process. Thus, the expected number of successes coming from cells in a given state and a certain voxel attempting a process can be drawn from a binomial distribution with a probability associated to the process. Following this elaboration, the number of cells successfully undergoing division, death, migration or transition to another state are calculated voxel-wise and state-wise at each time step by randomly sampling the corresponding binomial distribution B(*N*,*p*), whose *N* will be the number of cells in a given state within a voxel, and whose probability *p* will be the rate of the process modulated by the time step length Δt.

**Parameter estimation**

To run murine tumor simulations, a grid of 100 x 100 x 100 voxels was used, each voxel having a side length of 100 μm, and a carrying capacity *K* of 200 cells. Therefore, voxel volume was 10^-3^ mm^3^. A time step length of Δt = 15 minutes was chosen, in order to keep a good balance between typical cell dynamics times (proliferation and motility) and to be able to resolve the TMZ exponential decay times of about 2 hours. Simulations started with an initial inoculum in-silico of 3×10^5^ cells, and finished when tumors reached 20 mm^3^, in agreement with typical tumor volumes achieved by the time mice are sacrificed. Therapy was implemented to resemble the actual administration of drug to mice in the experimental part of this study. Three doses of TMZ were simulated in-silico, the first 7 days after initial inoculum. The remaining doses are administered with a gap of 1, 4, 7 or 13 days, depending on the dose spacing selected. TMZ pharmacokinetics were not modelled explicitly; instead, the ratio between current TMZ concentration and TMZ concentration achieving the maximum effect was used. This ratio can range between 0 and 1. TMZ has a plasma half-life of 1.8 hours, which is larger than the selected time step length, so TMZ concentration exponential decay over time can be resolved in the model.

To run human tumor simulations, again a grid of 100 x 100 x 100 voxels was used, but this time voxel side length was increased to 1 mm (with total volume being 1 mm^3^), in order to match typical voxel sizes of high-resolution magnetic resonance images. Carrying capacity was increased to 2 x 10^5^ cells. Time step length was set to Δt = 4 hours, to fasten simulation time while still keeping good resolution of human cell processes. During a 12-hour window starting from each TMZ dose administration, time step length was reduced to 15 minutes, to accurately resolve exponential drug decay. Simulations started with 10 PN cells placed in central voxel. The diagnostic volume of GBMs was randomly sampled from an empirical distribution of diagnostic sizes obtained from real patient data from TCIA. Unfortunately, an analogous empirical distribution of tumor volumes for sizes at death cannot be obtained, since the last available measurement corresponds to the last imaging follow-up, which does not match patient’s death. To address this issue, we fixed a maximum reachable tumor volume of 120 cm^3^ and simulations were stopped when tumors reached this size. Having defined diagnostic and death volumes in the context of the mathematical model, we could compute a survival time in-silico for every simulated tumor. This provided the required time points in order to run virtual clinical trials.

**Patients**

Volumetric data from a cohort of 69 patients of GBM from The Cancer Imaging Archive (TCIA-TCGA) was used to parametrize the model. Contrast-enhancing volume, necrotic volume and total volume were computed for each tumor in a previous work^5^. With these measures we built an empirical distribution of diagnostic sizes, that we used to random sample realistic diagnostic volumes for simulated tumors. This distribution was validated with independent patient data^6^, where a cohort of 209 GBM patients provided a median preoperative tumor volume of 24.9 cm^3^, and an interquartile range of 11.1-49 cm^3^, which agrees with our empirical distribution.

Mutation, CNV and immunohistochemical data from a cohort of 51 patients of GBM from the TOG study (Therapy Optimization in Glioblastoma) was also used to parametrize the model. TOG study was approved by the Institutional Review Board of all involved hospitals (Marqués de Valdecilla, Sanchinarro, 12 de Octubre, Virgen de la Salud, General Universitario de Ciudad Real, Universitario de Málaga, Manises, Universitario de Albacete). Selection criteria were lack of K27M mutation in H3F3A, lack of V600E mutation in BRAF, IDH1 wild-type tumors, and availability of Ki67 data.

**Virtual clinical trial**

To assess the effect that increasing dose spacing has on survival, we performed a virtual clinical trial with cohorts of simulated human glioblastomas treated under different conditions: control, standard therapy (TMZ during 5 days + 23 resting days), protracted therapies with progressively increasing resting periods, and low dose density therapies with progressively increasing intervals between doses (without resting periods). In standard therapy, 6 cycles were administered, meaning 30 TMZ doses were given. Treatment started 1 week after diagnosis. In all protracted therapies, these 30 doses were spaced either by increasing resting period (but keeping an interval of 1 day between doses), or by increasing interval between doses. We evaluated both fast-growing glioblastomas and slow-growing glioblastomas. 50 tumors were simulated for each branch, so the whole virtual clinical trial comprises 600 simulated tumors. Differences in survival between branches were computed by comparing estimated Kaplan-Meier curves with log-rank test. The median survival differences were also computed for each therapy scheme against control, to assess their effectiveness.

**Parameter analysis of discrete mathematical model**

A two-level parameter exploration was performed with the discrete mathematical model. On the first level, we run virtual murine tumor simulations varying μ_sq_ and μ_qs_. In this way, we generated a cohort of tumors with different Ki67 levels and OS, including fast-growing and slow-growing GBM models. On the second level, we explored the effect of three key parameters: μ_PT_, μ_ps_ and R_fac_. μ_PT_ affects the spontaneous transition from PN to MES cells, thus influencing the time it takes for the PN-MES phenotypic transition to occur within the tumor. μ_ps_ determines the time persister cells can withstand the lack of TMZ before turning back to PNs cells. R_fac_ indicates the ratio between dying fractions of both MES and PN cells when treated when TMZ; hence, a greater R_fac_ means a lower resistance of MES cells compared to PN ones. Considering both levels of the parameter exploration, we generated three cohorts of virtual murine tumors, one per parameter evaluated on the second level. Each tumor was characterized by a triad of μ_sq_, μ_qs_ and μ_PT_/μ_ps_/R_fac_ values. The ranges of values selected for each parameter can be seen at Supplementary Table S2. Each virtual tumor was simulated five times with the same random number generator seed. In this way, we could isolate the effect of TMZ on survival and MES content, without any other stochastic elements influencing the outcome. More specifically, virtual ‘twinned’ tumors were simulated without treatment (control), and with 1-, 4-, 7- and 13-day spacing.

On the first level of parameter exploration, we reproduced real murine tumor dynamics in terms of OS, Ki67 and PN-MES transition. Parameters μ_sq_ and μ_qs_ were chosen according to a Bayesian criterion. After running several simulations without treatment, those whose initial parameters produced realistic murine tumors (with OS ranging between 30 and 60 days, and Ki67 levels ranging from 5 to 30%) were selected and used to select plausible ranges for these two parameters. This is a rough but effective approach to ABC rejection algorithm. Initial μ_sq_ ranged from 1 to 0.33 days^-1^, while initial μ_qs_ ranged from 0.1 to 0.0166 days^-1^ (Supplementary Figure S9). Tumors with Ki67 levels around 30% have associated an OS of 30 days, thus encompassing the fast-growing group. Meanwhile, tumors with Ki67 around 5% have associated an OS of 60 days, hence belonging to the slow-growing group. Treatment improved survival and reduced MES tumor content, independently of dose spacing.

On the second level of parameter exploration, we observed that increasing the value of μ_PT_ reduces the amount of MES cells in the tumor (in absence of treatment). This effect is more noticeable in fast-growing tumors. When applying TMZ, both 4-day and 13-day spacings showed an increase in MES cells compared to control, being more pronounced for larger values of μ_PT_. However, the increase was smaller for the 13-day spacing (10% more MES cells than control) compared to the 4-day spacing (20% more MES cells than control). Interestingly, some treated slow-growing tumors showed a decrease in MES cells compared to control.

When looking at R_fac_, we observed that, if MES cells are as resistant as PN cells (Rfac = 1), fast-growing tumors are the most benefited from spacings, with the 4-day spacing showing the greatest effect. If MES cells are fully resistant (Rfac = 0), then slow-growing tumors are the most benefited by the spacings, with almost no differences between both 4-day and 13-day spacings. None of these cases are realistic, but they help us narrowing down the behavior it should be expected from a tumor depending on their MES cell content.

Finally, the analysis of μ_ps_ revealed that, for short persistence times, the benefit from spacing is just moderate. However, for long persistence times, OS increase of treated tumors becomes greater, especially for slow-growing ones. Regarding resistance, the greater μ_ps_ is, the larger the increase in MES cells is. 13-day spacing produced a smaller increase in MES cells than 4-day spacing, suggesting that short cycles may be worse when it comes to the emergence of resistance.

**Virtual human tumor kinetics**

Besides the rate of cell division, there are two parameters that govern the growth kinetics of the simulated tumors: μ_sq_ and μ_qs_. Their interaction determines the balance between quiescent and proliferative cells that will exist within the tumor, thus influencing the proportion of the latter (i.e. Ki67 LI), and global tumor growth rate, ultimately impacting overall survival (OS). We used a Bayesian inference approach with a thorough parameter search to identify the pairs of parameters that produce realistic tumors in terms of both Ki67 LI and OS. In this study, we classified GBMs in two groups according to their proliferation level: a fast-growing group of virtual GBMs, represented by a median OS of approximately 5 months and a median Ki67 LI of 25%; and a slow-growing group of virtual GBMs, which we represented with a median Ki67 LI of approximately 2.5%, and a median OS of approximately 15 months. Note that these OS are estimated for untreated tumors that did not underwent total nor partial surgical resection. We established ranges of μ_sq_ and μ_qs_ values and paired them to build a grid, where each point on the mesh corresponds to a pair of parameters μ_sq_ and μ_qs_. More specifically, parameters ranged from 0.45 to 2.5 days^-1^. We ran simulations for each grid point, so that we could select the grid points yielding realistic tumors in terms of Ki67 LI and OS. The same random number generation seed was used for all simulations. As a result, observable effects can only be ascribed to the initial parameter choice.

Once every simulation from every grid point had associated values of Ki67 LI and OS, we calculated an interpolation surface (spline method using MATLAB’s *griddedInterpolant* function) to improve predictive resolution. Later, we extrapolated (linear method using MATLAB’s *griddedInterpolant* function) Ki67 LI and OS values over a wider grid of parameters to increase the predictive power of these simulations. This provided an estimate of Ki67 and OS values for grid points not taken into account in the initial grid definition (Supplementary Figure S10A for OS, and Supplementary Figure S10B for Ki67). Then, the grid locations with Ki67 and OS values that matched the previously described tumor categories were chosen (Supplementary Figure S10C). The starting parameters μ_sq_ and μ_qs_ that produce genuine OS and Ki67 are represented by these points (Supplementary Figure S10D). Using these ranges, we random sample initial values of μ_sq_ and μ_qs_ for virtual human tumors used in the virtual clinical trial.

**REFERENCES**

1. Gargini R, Segura-Collar B, Herranz B, et al. The IDH-TAU-EGFR triad defines the neovascular landscape of diffuse gliomas. *Sci Transl Med.* 2020; 12(527).

2. Segura-Collar B, Garranzo-Asensio M, Herranz B, et al. Tumor-derived pericytes driven by EGFR mutations govern the vascular and immune microenvironment of gliomas. *Cancer Res.* 2021.

3. Livak KJ, Schmittgen TD. Analysis of relative gene expression data using real-time quantitative PCR and the 2(-Delta Delta C(T)) Method. *Methods*. 2001; 25(4):402-408.

4. Jiménez-Sánchez J, Martínez-Rubio Á, Popov A, et al. A mesoscopic simulator to uncover heterogeneity and evolutionary dynamics in tumors. *PLoS Comput Biol*. 2021; 17(2): e1008266.

5. Pérez-Beteta J, Molina-García D, Ortiz-Alhambra JA, et al. Tumor Surface Regularity at MR Imaging Predicts Survival and Response to Surgery in Patients with Glioblastoma. *Radiology*. 2018; 288(1):218-225.

6. Bette S, Barz M, Wiestler B, et al. Prognostic Value of Tumor Volume in Glioblastoma Patients: Size Also Matters for Patients with Incomplete Resection [published correction appears in *Ann Surg Oncol*. 2018;25(Suppl 3):989]. *Ann Surg Oncol*. 2018; 25(2):558-564.

7. Rabé M, Dumont S, Álvarez-Arenas A, et al. Identification of a transient state during the acquisition of temozolomide resistance in glioblastoma. *Cell Death Dis.* 2020; 11(1):19.

8. Sharma SV, Lee DY, Li B, et al. A chromatin-mediated reversible drug-tolerant state in cancer cell subpopulations. *Cell.* 2010; 141(1):69-80.

9. He Y, Kaina B. Are There Thresholds in Glioblastoma Cell Death Responses Triggered by Temozolomide?. *Int J Mol Sci*. 2019; 20(7):1562.

**
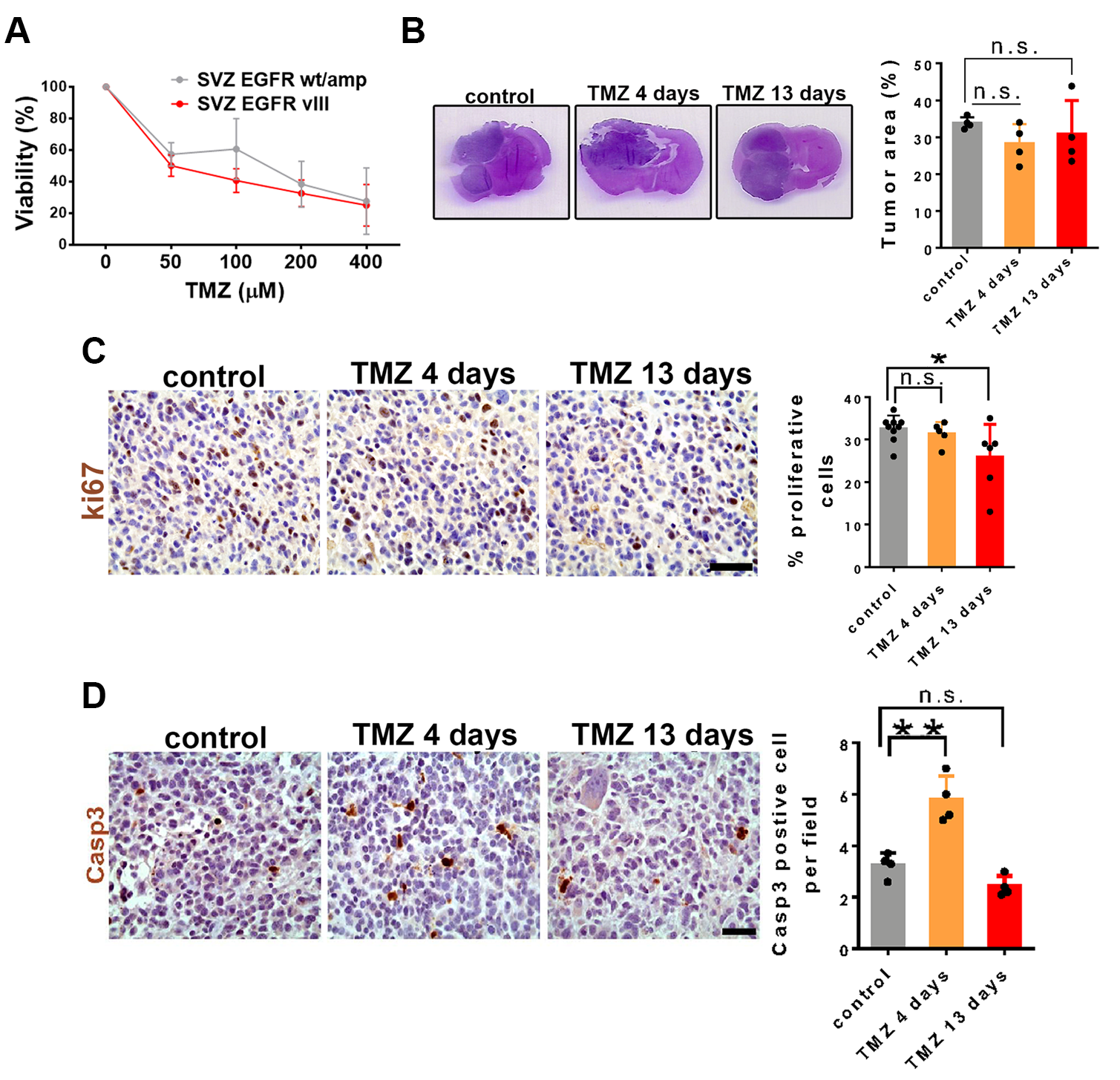
**

**Supplementary Fig. S1.** Effect of increasing spacing between TMZ doses in SVZ EGFR wt/amp tumors. (A) SVZ cells (EGFRwt or EGFRvIII) were incubated in the presence of increasing amounts of TMZ for 3 days and viability was measured with Alamar Blue. Percentage of survival is shown in the graphic (n=3). (B) Representative histological images stained with hematoxylin and eosin (H&E) from coronal brain sections of SVZ EGFR wt/amp tumors from (Fig. 2) of control, TMZ 4 days and TMZ 13 days treatment condition. Quantification of tumor area percentage is shown on the right (n=3). (B) Representative pictures of immunohistochemical (IHC) staining of ki67 in the same tumors from (A) and quantification of the percentage of proliferative cells on the right (n=3). (C) Representative pictures of IHC staining of Active Caspase3 in SVZ EGFR wt/amp in the same tumors from (A). Quantification is shown on the right (n=3).*P ≤ 0.05, n.s.=non significant.


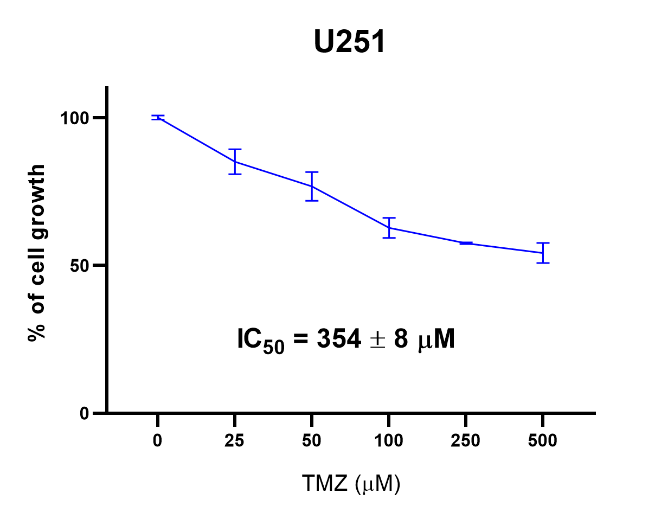


**Supplementary Fig. S2.** Response of U251 cells to TMZ**.** U251 cells were treated with different concentrations of TMZ for 3 days and their viability was analyzed by MTT. The percentages of cell growth shown in the graphic are from three experiments (n=3). The IC_50_ was determined by nonlinear regression using GraphPad Prism 8.0.2.


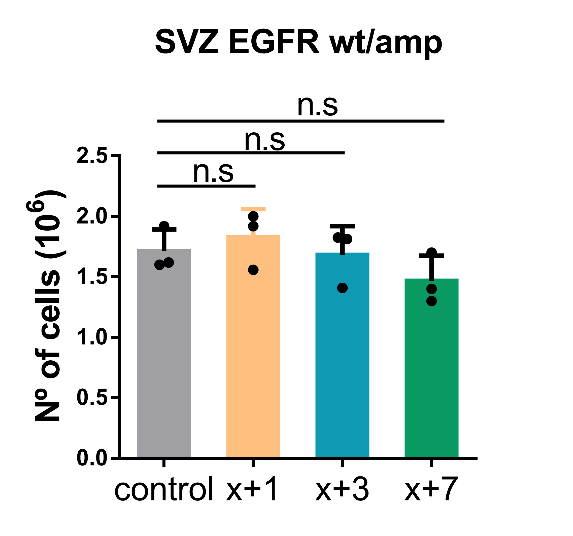


**Supplementary Fig. S3.** Response of SVZ glioma cells to TMZ in vitro**.** Quantification of the number of cells in SVZ EGFR wt/amp after TMZ treatment schedule (n=3).





**Supplementary Fig. S4.** Induction of persister genes in SVZ-EGFRvIII cells. SVZ-EGFR vIII cells were treated in-vitro with different TMZ schedules: control, TMZ 1 day, TMZ 3 days and TMZ 7 days. The graph shows the qRT-PCR analysis of different persister genes. *Actin* was used for normalization (n=3).


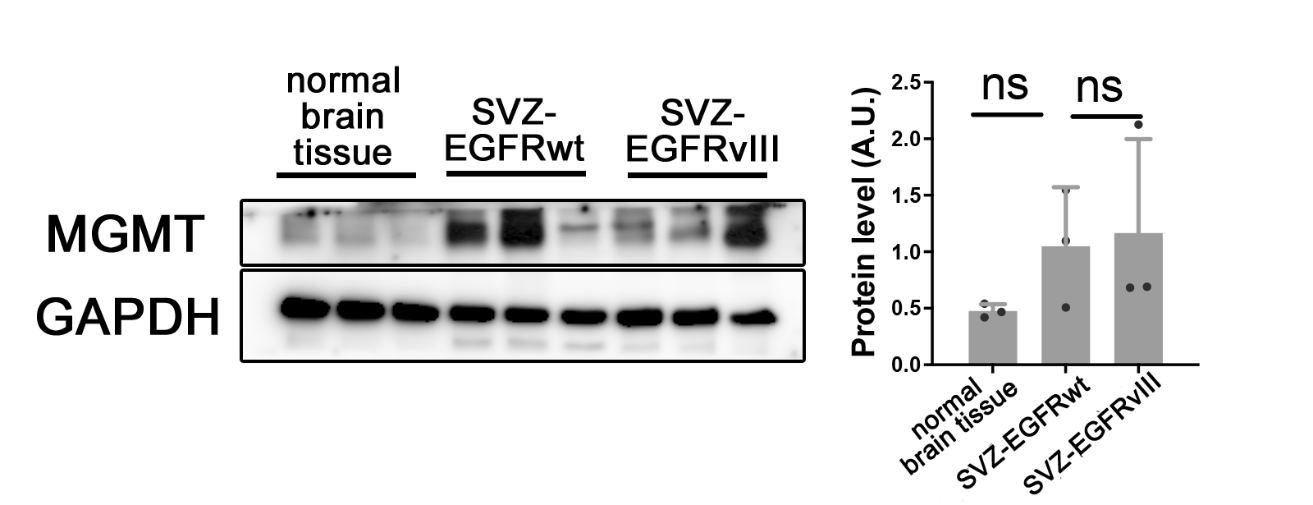


**Supplementary Fig. S5.** Analysis of MGMT expression in SVZ tumors. Proteins were extracted from normal Nude mouse brains and SVZ-EGFRwt or SVZ-EGFRvIII tumors and a Western Blot analysis was performed. GAPDH was used as a loading control. Quantification is shown on the right.





**Supplementary Fig. S6.** Effect of TMZ *in-vitro* in 2D cultures. (A) TMZ treatment scheme in long-term 2D cell cultures of U251 glioblastoma cell line. (B) Doubling Time (dt) calculated using RTCA 1.2.1 software illustrates the anti-proliferative effects of TMZ treatments in U87 cell line. (C) Relative gene expression levels of *ABCB1* and *MGMT* in U251 cells obtained by qRT-PCR. All values are expressed as mean ± SD and *ACTB* was used for normalization (n = 3).


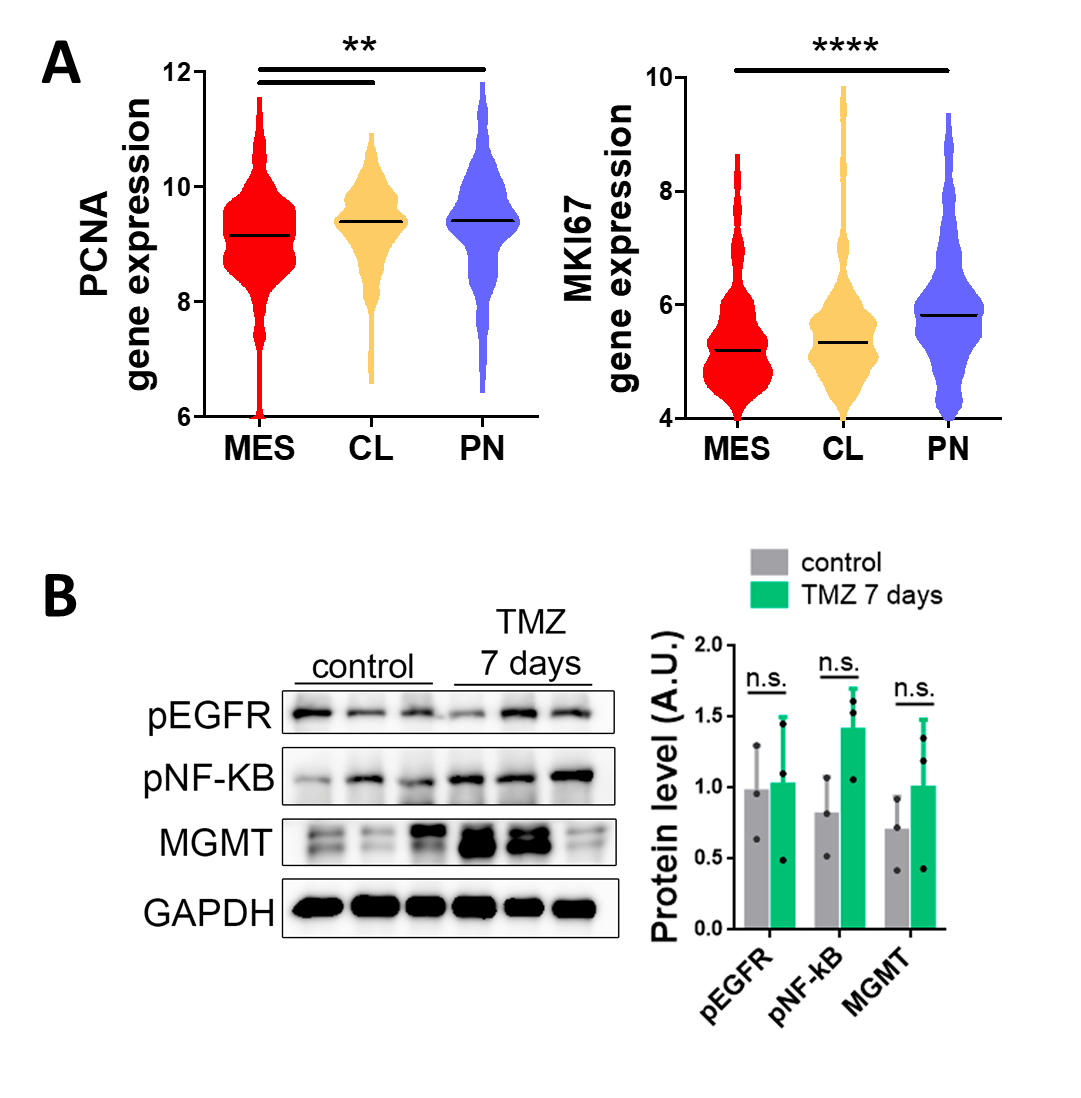


**Supplementary Fig. S7.** Expression of proliferation-related markers in different subgroups of gliomas. (A) RNA-seq analysis of *PCNA* (left) and *MKI67* (right) in a TCGA cohort stratified in to three groups: mesenchymal (MES), classical (CL) and proneural (PN) tumors. (B) Western blot analysis of phosphorylated EGFR (pEGFR) and NF-kB (p65) (pNF-kB) in SVZ-EGFRvIII tumors from Fig. 2. GADPH was used as a loading control. Quantification is shown on the right (n= 3).


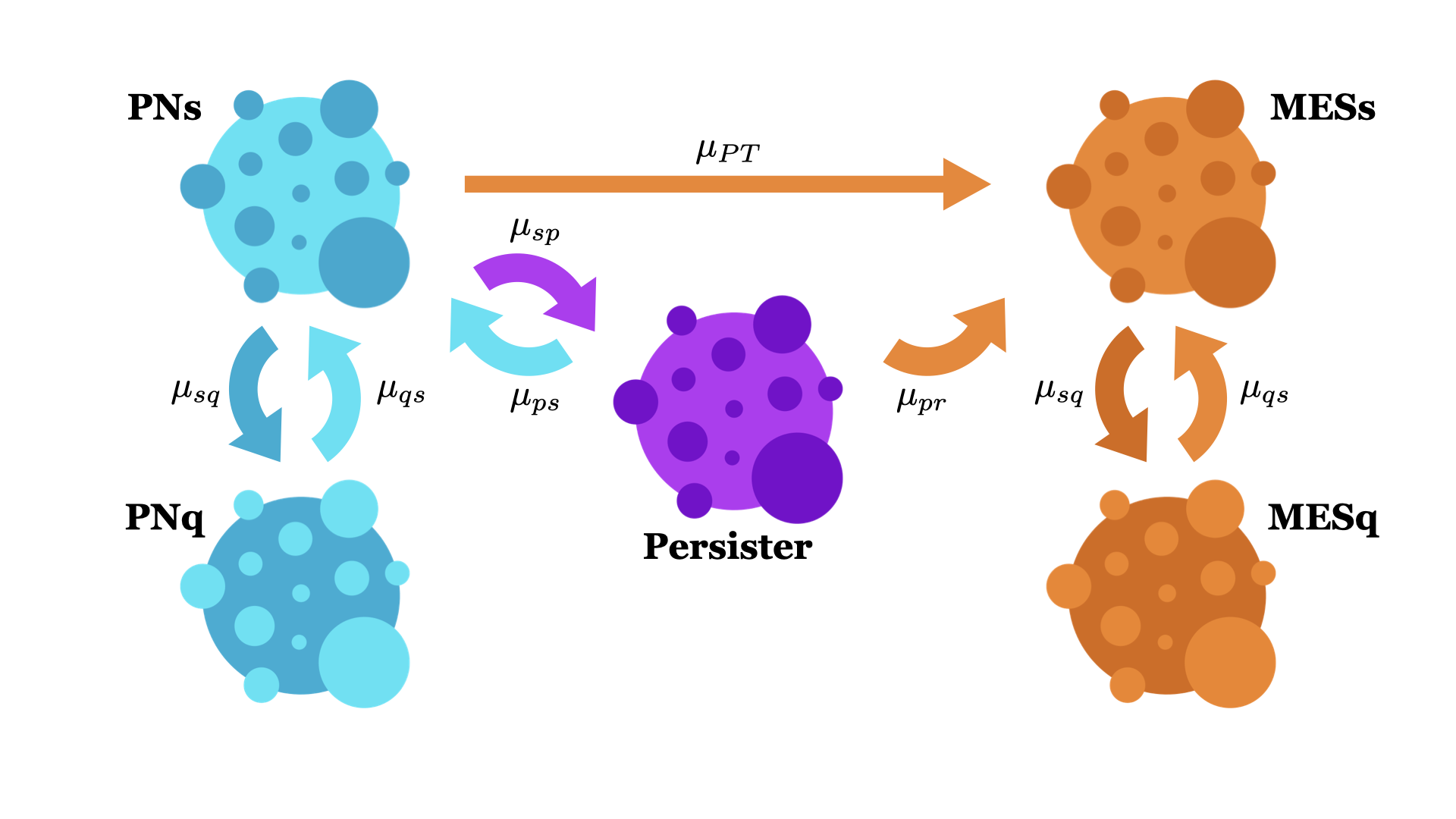


**Supplementary Figure S8.** Allowed cell transitions in the model. Both PN and MES cells share the same proliferative-quiescent dynamics. PNs can become MES either directly or due to TMZ exposure. In the latter case, the transition occurs through a transient reversible persister state.


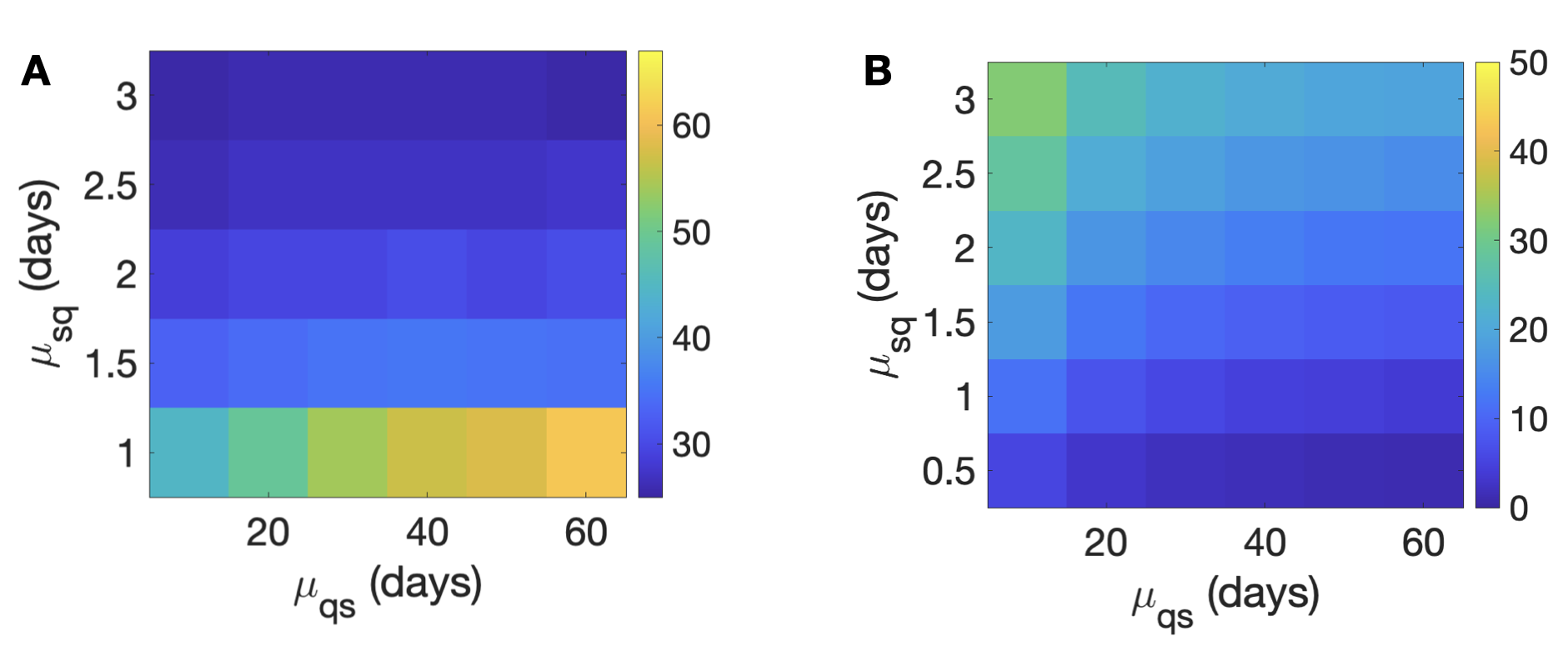


**Supplementary Fig. S9.** Overall survival in days (**A**) and Ki67 % (**B**) obtained from different combinations of initial ranges of parameters μ_sq_ and μ_qs_ for murine tumor simulations.

 **Supplementary Fig. S10.** Parameter search performed to fit the model to realistic fast- and slow-growing glioblastomas. (**A)** Extrapolation surface providing OS estimates depending on $\mu_{sq}$ and $\mu_{qs}$ values, based on produced simulation data. (**B)** Extrapolation surface providing Ki67 LI estimates. (**C)** Selected $\mu_{sq}$ and $\mu_{qs}$ values that provide realistic fast- and slow-growing GBMs. (**D)** Ki67 and OS predicted by the selected $\mu_{sq}$ and $\mu_{qs}$ values.
