# Supplementary material for: On optimal temozolomide scheduling for slowly growing glioblastomas
Source: Neurooncol Adv. 2022 Sep 27;4(1):vdac155. doi: 10.1093/noajnl/vdac155 (PMC9616068; doi:10.1093/noajnl/vdac155)
Supplement: vdac155_suppl_Supplementary_Table_S1 [file vdac155_suppl_supplementary_table_s1.docx]

**Supplementary Table S1.** Primers used for the qRT-PCR assays.

| **Gene** | **Forward (5'-3´)** | **Reverse (5'-3´)** |
| --- | --- | --- |
| **MGMT** | GCAATTAGCAGCCCTGGCA | CACTCTGTGGCACGGGAT |
| **ABCB1** | CCCATCATTGCAATAGCAGG | GTTCAAACTTCTGCTCCTGA |
| **CHI1L3** | CCCTGGACGGAGAGACAAAC | GCCTCAACATGTACCCCACA |
| **HB-EGF** | TGTCTGTCTGCTGGTCATCG | ATGGGAGGCCCAATCCTAGA |
| **ACTB** | TGGACATCCGCAAAGACCTGTAC | TCAGGAGGAGCAATGATCTTGA |
| **Mgmt** | CTCGTGCAGTAGGAGGAGCAAT | AGAAGCCACTCCTTCACAGCCT |
| **Fat2** | AGAATCACGGCGTCAGATGGCA | TCACGGACCTTGCCAGTGTAGA |
| **GAPDH** | GCTTTGCCAACATCAGCAGCGA | GGAGGGTCTTCAGGTTGGTGT |
| **Chi3l1** | CGCTACTTGGAAGTATCAGAGCC | TGGTGACAGTGTGGAAGATGCG |
| **Hb-egf** | GAGTTCCGTACTCCCTCTTGCA | AGCCAAGACTGTAGTGTGGTC |
| **Actin** | CATTGCTGACAGGATGCAGAAGG | GCTGGAAGGTGGACAGTGAGG |
| **Serpine1** | CCTCTTCCACAAGTCTGATGGC | GCAGTTCCACAACGTCATACTCG |
| **Cd44** | CGGAACCACAGCCTCCTTTCAA | TGCCATCCGTTCTGAAACCACG |
| **Timp1** | TCTTGGTTCCCTGGCGTACTCT | GTGAGTGTCACTCTCCAGTTTGC |
| **Mash1** | CGGAACTGATGCGCTGCAAACG | GGCAAAACCCAGGTTGACCAAC |
| **Olig2** | ATGCACGACCTCAACATCGCCA | ACCAGTCGCTTCATCTCCTCCA |
| **Sox2** | AACGGCAGCTACAGCATGATGC | CGAGCTGGTCATGGAGTTGTAC |
