# Supplementary material for: On optimal temozolomide scheduling for slowly growing glioblastomas
Source: Neurooncol Adv. 2022 Sep 27;4(1):vdac155. doi: 10.1093/noajnl/vdac155 (PMC9616068; doi:10.1093/noajnl/vdac155)
Supplement: vdac155_suppl_Supplementary_Table_S2 [file vdac155_suppl_supplementary_table_s2.docx]

**Supplementary Table S2.** Model parameters used to run murine and human simulations.

| **Parameter** | **Meaning** | **Value (mouse)** | **Value (human)** | **Units** | **Reference** |
| --- | --- | --- | --- | --- | --- |
| **τ_p_** | Division time (proliferative cells) | 19 | 48-72 | h | Estimated from [7] |
| **ρ_m_** | Migration coefficient | 0.12 | 0.4 | mm^2^/day | Estimated from [7] |
| **τ_d_** | Death time | 100 | 288-312 | h | Estimated from [7] |
| **μ_sq_** | Transition rate from proliferative state to quiescent | 0.3333-1 | 0.2-0.6 | day^-1^ | Estimated from [7] |
| **μ_qs_** | Transition rate from quiescent state to proliferative | 0.0166-0.1 | 0.03-0.2 | day^-1^ | Estimated from [7] |
| **μ_sp_** | Transition rate from PNs to persister | 0.0333 | 0.0333 | min^-1^ | Estimated from [8] |
| **μ_ps_** | Transition rate from persister to PNs | 0.2 | 0.2 | day^-1^ | Explored in this work |
| **μ_pr_** | Transition rate from persister to MESs | 0.0111 | 0.0111 | min^-1^ | Estimated from [8] |
| **μ_PT_** | Transition rate from PNs to MESs | 0.0208-0.0833 | 0.0208 | h^-1^ | Explored in this work |
| **S_f_** | Survival fraction after TMZ dosing | 0.75 | 0.5 | unitless | Estimated from [9] |
